# Defect Chemistry of Spinel Cathode Materials—A Case Study of Epitaxial LiMn_2_O_4_ Thin Films

**DOI:** 10.1021/acs.chemmater.3c00814

**Published:** 2023-06-29

**Authors:** Andreas E. Bumberger, Christin Boehme, Joseph Ring, Sergej Raznjevic, Zaoli Zhang, Markus Kubicek, Juergen Fleig

**Affiliations:** † Institute of Chemical Technologies and Analytics, TU Wien, Vienna 1060, Austria; ‡ Erich Schmid Institute of Materials Science, Leoben 8700, Austria

## Abstract

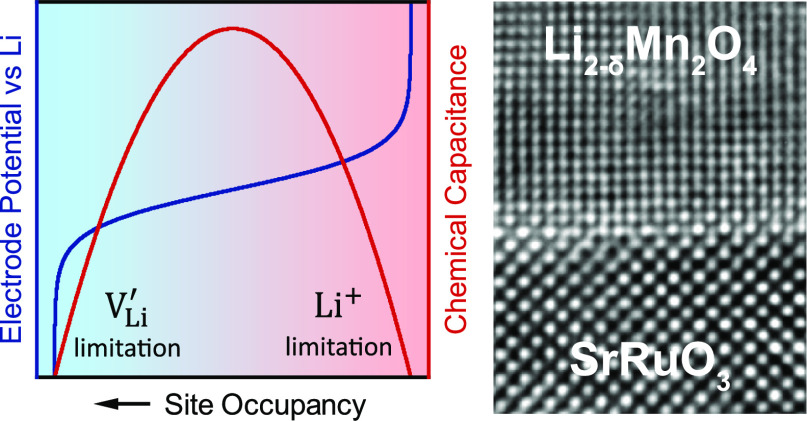

Spinels of the general formula Li_2–δ_M_2_O_4_ are an essential class of cathode materials for Li-ion batteries, and their optimization in terms of electrode potential, accessible capacity, and charge/discharge kinetics relies on an accurate understanding of the underlying solid-state mass and charge transport processes. In this work, we report a comprehensive impedance study of sputter-deposited epitaxial Li_2–δ_Mn_2_O_4_ thin films as a function of state-of-charge for almost the entire tetrahedral-site regime (1 ≤ δ ≤ 1.9) and provide a complete set of electrochemical properties, consisting of the charge-transfer resistance, ionic conductivity, volume-specific chemical capacitance, and chemical diffusivity. The obtained properties vary by up to three orders of magnitude and provide essential insights into the point defect concentration dependences of the overall electrode potential. We introduce a defect chemical model based on simple concentration dependences of the Li chemical potential, considering the tetrahedral and octahedral lattice site restrictions defined by the spinel crystal structure. The proposed model is in excellent qualitative and quantitative agreement with the experimental data, excluding the two-phase regime around 4.15 V. It can easily be adapted for other transition metal stoichiometries and doping states and is thus applicable to the defect chemical analysis of all spinel-type cathode materials.

## Introduction

Cathode materials for Li-ion batteries (LIBs) have become one of the most essential classes of modern-day functional materials. Their optimization in terms of capacity, operating voltage, cycling stability, safety, and rate capability is a key part of the collective effort to max out the overall technological potential of LIBs for various applications. Given the rapid improvements of energy densities over the last few years, focus is increasingly put on charging speed and discharge power density. Thus, more than ever, there is a need for a detailed understanding of the individual mass and charge transport processes that determine the overall kinetics. A major challenge in this pursuit is posed by the morphological and compositional complexity of porous electrodes, which makes it difficult to separate the individual contributions of pore diffusion, interfacial charge transfer, and solid-state diffusion. To further complicate things, the active material in porous electrodes is usually processed in the form of secondary agglomerates, rather than single crystallites, such that boundaries between primary particles may heavily impact the observed solid-state kinetics. Moreover, the active particles in porous electrodes are often (partly) coated, for example, with carbon.

One way to isolate the intrinsic bulk transport properties of a given active material is the fabrication and characterization of thin-film electrodes exhibiting a well-defined geometry and composition. As previously exemplified for Li_1–δ_CoO_2_,^1^ this approach allows the evaluation of ionic conductivity, chemical capacitance, and chemical diffusivity as a function of state-of-charge (SOC) from comparatively simple impedance models that describe solid-state chemical transport as the resistive and capacitive interplay of ions and electrons based on the Nernst–Planck-equation. This set of elementary material properties fully describes the transport of mass and charge within a single crystallite and can be related back to defect chemical principles and defect thermodynamics. If these bulk material properties and their continuous variation with the SOC are available, they can be used as input parameters for more complex models considering the behavior of the active material in the intricate network of a porous LIB electrode.

LiMn_2_O_4_ (LMO) of the space group Fd3̅m is the prototypical spinel cathode material. Its crystal structure is commonly described as a cubic close packing of 32 O atoms per unit cell, where Mn occupies half of the 32 octahedral sites (16d sites) and Li occupies one eighth of the 64 tetrahedral sites (8a sites). The unit cell thus consists of 32 O atoms, 16 Mn atoms, and 8 Li atoms, with Mn being in the mixed valence state of +3.5.

Upon oxidation of Mn^3+^ in Li_2–δ_Mn_2_O_4_ to a valence state of +4 (δ > 1), Li^+^ is released from the occupied tetrahedral 8a sites. The corresponding transition from LiMn_2_O_4_ to λ-Mn_2_O_4_ (both of space group Fd3̅m) proceeds in two main stages, as evidenced by the characteristic double plateau of the charge curve around 4.00 and 4.15 V versus Li^+^/Li. The first plateau is commonly described as a single-phase solid solution of the general composition Li_2–δ_Mn_2_O_4_ (δ > 1) up to a nonstoichiometry value of δ = 1.5, accompanied by a gradual decrease of the cubic lattice parameter from 8.24 to 8.19 Å.^2^ Depending on synthesis conditions and Li/Mn stoichiometry, this storage regime has also been reported to partially involve the coexistence of two structurally very similar phases, often visible as a sharp peak superimposed on the broader solid-solution peak in differential capacity curves.^3^ At δ = 1.5, the remaining Li ions are ordered in a way that minimizes electrostatic repulsion and thus stabilizes the occupied 8a sites with respect to the now emptied 8a sites.^4−7^ As a result, further delithiation from the occupied Li sites occurs at a higher electrode potential and leads to a sudden drop of the lattice parameter down to 8.14 Å.^2^ In Li_0.5_Mn_2_O_4_, the formerly equivalent 8a sites are thus split into two nonequivalent tetrahedral sites, one being fully occupied and the other being empty. In addition, it has been proposed that Mn^3+^/Mn^4+^ ordering according to Li_0.5_Mn_0.5_^3+^Mn_1.5_^4+^O_4_ could occur analogously to the well-established Ni^2+^/Mn^4+^ ordering observed in the isostructural high-voltage spinel LiNi_0.5_Mn_1.5_O_4_ (LNMO).^8−12^ The second plateau, spanning from δ = 1.5 to δ = 2 with a significantly flatter potential profile than the first plateau, has been shown to involve a first-order phase transition from a Li-rich (δ ≈ 1.65) to a Li-poor (δ ≈ 1.9) spinel phase with lattice parameters of approximately 8.14 and 8.04 Å, respectively.^2^

On the other hand, Mn^4+^ in Li_2–δ_Mn_2_O_4_ (δ < 1) can also be reduced to a valence state of +3, accompanied by the insertion of Li^+^ into the remaining octahedral sites up to a final stoichiometry of Li_2_Mn_2_O_4_ (t-LiMnO_2_). This insertion process is known to proceed as a first-order phase transition from the cubic Fd3̅m to the tetragonal I4_1_/amd phase at a potential of approximately 2.89 V versus Li^+^/Li, as Mn^3+^:Mn^4+^ ratios above 1 induce a Jahn–Teller distortion in the cubic host lattice.^5,13−15^ Since a large part of the analysis presented in this work requires the presence of a single-phase solid solution, this low-potential plateau is not further considered.

In total, electrochemical Li storage in LMO is therefore divided into three separate regimes: (i) storage at octahedral 16d sites (0 ≤ δ ≤ 1), (ii) disordered storage at tetrahedral 8a sites (1 ≤ δ ≤ 1.5), and (iii) ordered storage at tetrahedral 8a sites (1.5 ≤ δ ≤ 2). Due to the effective nonequivalence of the tetrahedral sites, they will be referred to as T1 (1 ≤ δ ≤ 1.5) and T2 (1.5 ≤ δ ≤ 2) sites in the following. Leaving aside the possibility of Mn^3+^/Mn^4+^ ordering, all three storage regimes involve the same redox couple with fully equivalent electronic lattice positions for all three storage regimes (0 ≤ δ ≤ 2).

In this work, we present a comprehensive impedance study of sputter-deposited epitaxial Li_2–δ_Mn_2_O_4_ thin films on SrRuO_3_ (SRO) over the entire high-voltage SOC range (3.7–4.4 V versus Li metal, approximately corresponding to 1 ≤ δ ≤ 2) in fine potential increments of 10 mV. We deduce a complete set of bulk electrochemical properties, consisting of the area-specific charge-transfer resistance, ionic conductivity, chemical capacitance, and chemical diffusivity as a function of SOC. Finally, we provide a defect chemical model and Brouwer diagram for LMO that consistently describes the observed trends in terms of Li chemical potential, Li activity, and point defect concentrations. The proposed model can easily be adapted for other transition metal stoichiometries and therefore paves the way toward a more detailed understanding of the defect chemistry of all spinel cathode materials.

## Experimental Section

### Sample Preparation

**Figure 1:**
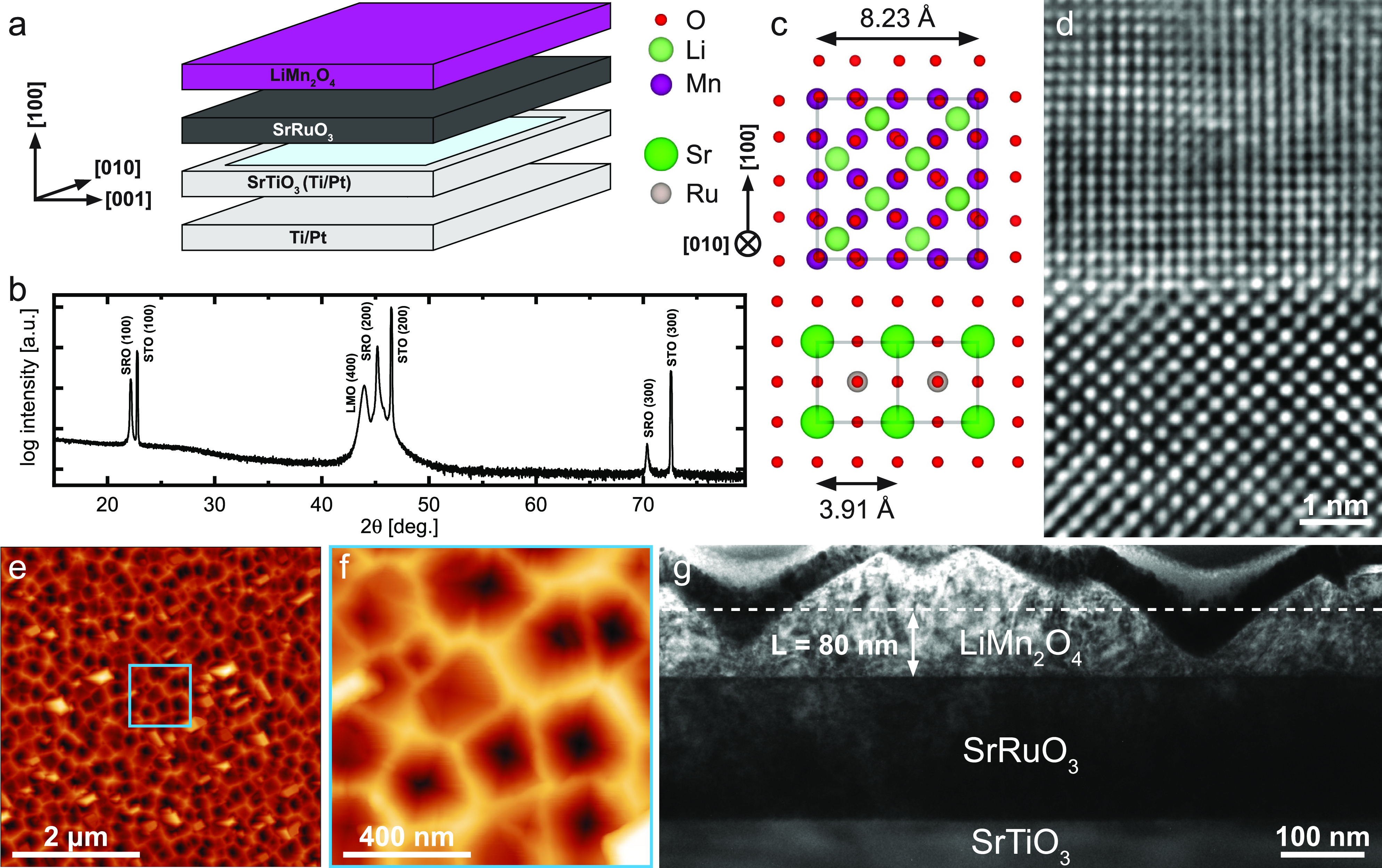
(a) Schematic representation of a typical sample, consisting of a (100)-oriented polished STO single crystal coated with a bilayer of Ti/Pt on the backside and around the edges, an epitaxial SRO thin-film current collector and epitaxial LMO thin film. (b) θ-2θ X-ray diffractogram showing the (h00) reflexes of LMO and SRO, suggesting the presence of an epitaxial LMO/SRO bilayer on the STO (100) substrate. (c) Atomic representation of the (200)_SRO_//(400)_LMO_ epitaxial relationship, which is confirmed by the high-resolution TEM image of the LMO (top)/SRO (bottom) interface in panel d. For SRO, the in-plane lattice parameter was confirmed by reciprocal space mapping (Figure S1). For LMO, strain relaxation is assumed, resulting in the in- and out-of-plane lattice parameters both being identical to the bulk value of 8.23 Å. (e, f) Atomic-force microscopy images of the LMO thin-film surface, showing the characteristic pyramidal morphology of a (400)-oriented spinel thin film. (g) Bright-field TEM image showing an average LMO film thickness of about 80 nm.

Epitaxial thin films of SrRuO_3_ (SRO) and LiMn_2_O_4_ (LMO) were deposited via radio-frequency (RF) magnetron sputtering onto polished SrTiO_3_ (STO) (100) single-crystal substrates (10 × 10 × 0.5 mm^3^, MaTecK, Germany) in a custom-built deposition chamber (Huber Scientific, Austria). Sputter targets of LMO and SRO with a diameter of 2″ were obtained from ALB Materials, USA, and AEM Deposition, China, respectively, and abraded with sandpaper before each use to ensure a constant target stoichiometry for successive depositions. Substrates were sonicated in a 3% aqueous solution of Extran (Merck, Germany), bidistilled water and ethanol for 10 min per step prior to use. To provide an electronic contact to the backside for electrochemical measurements, a thin film of Ti/Pt (5/100 nm) was deposited onto the sides and edges of the substrates via DC sputtering at room temperature under an Ar atmosphere of 0.7/2.5 Pa and a current density of 5 mA/cm^2^. Ti is used to improve adhesion to the STO substrate, and for the given preparation procedure, we did not observe contact problems of the Ti/Pt layers. Subsequently, SRO and LMO were deposited at a substrate-to-target distance of 6.0 cm, a pressure of 2.5 Pa (25% O_2_, 75% Ar), a power of 60 W, and nominal substrate temperatures of 650 and 550 °C, respectively. The nominal substrate temperature on the heating stage was determined from a power-temperature calibration on a Y:ZrO_2_ (100) single-crystal (9.5 mol % Y_2_O_3_, CrysTec, Germany) of identical dimensions using an optical pyrometer and assuming a surface emissivity coefficient of ε = 0.9. The SRO thin films reported in this work had a thickness of approximately 170 nm as measured by TEM, corresponding to a deposition rate of 1.87 nm/min. For the textured LMO thin film, an average of approximately 80 nm was determined by TEM (cf. Figure 1g), corresponding to a deposition rate of 0.89 nm/min. Finally, the backside of the samples was covered with another sputter-deposited thin film of Ti/Pt (5/100 nm) to provide a good electrical contact to the steel plunger of the test cell. For the experiments presented in this work, two separate, nominally identical samples were prepared: one for structural characterization and one for electrochemical measurements.

### Structural Characterization

The as-prepared samples were characterized by means of X-ray diffraction (XRD), atomic force microscopy (AFM), and transmission electron microscopy (TEM). Out-of-plane θ-2θ diffractograms were acquired for 2θ angles of 10°–90° on an Empyrean X-ray diffractometer (Malvern Panalytical, UK) using a hybrid Kα monochromator of type 2XGe(220) on the incident beam side and a GaliPIX3D area detector in scanning line mode on the diffracted beam side. AFM images of the sample surface were recorded on a Nanoscope V multimode setup (Bruker) and analyzed using Gwyddion.^16^ An electron-transparent lamella for TEM imaging was prepared via standard lift-out techniques on a Thermo Fisher Scios 2 DualBeam FIB/SEM, operating with a Ga-ion beam at 30 kV accelerating voltage. After thinning at 30 kV, a final low-voltage cleaning step was performed at 5 and 2 kV to reduce the extent of superficial amorphization on the lamella. All TEM measurements were carried out on a JEOL JEM-2100F field-emission gun microscope equipped with an image-side spherical aberration corrector, operating at an accelerating voltage of 200 kV. TEM images were recorded using a Gatan Orisu SC1000 CCD camera. High-resolution TEM images were post-processed using an average background subtraction filter (ABSF).

### Electrochemical Characterization

For electrochemical measurements, a thin-film sample was transferred into an argon-filled glovebox (O_2_, H_2_O < 0.1 ppm) and assembled into a three-electrode test cell (PAT-Cell, EL-CELL, Germany) with a concentric Li reference ring electrode (EL-CELL), a Li counter electrode (ca. 10 × 10 × 0.6 mm^3^, Goodfellow, Germany), a glass fiber separator (260 μm, EL-CELL), and 80 μL of standard organic electrolyte (1 M LiPF_6_ in a 1:1 mixture of ethylene carbonate and dimethyl carbonate, Aldrich, USA). All electrochemical measurements were carried out at room temperature on a Biologic SP200 potentiostat with a built-in impedance analyzer. Cyclic voltammetry was performed at a scan rate of 1 mV/s in the working electrode potential range of 3.7–4.4 V versus Li^+^/Li. Potential-controlled impedance spectra (200 kHz–10 mHz, 6 points per decade) were recorded for the same voltage range in intervals of 10 mV using a perturbation amplitude of 10 mV. Before each impedance measurement, the working electrode was left to equilibrate for 5 min at the given potential.

## Results and Discussion

### Epitaxial LMO/SRO Thin Films

Figure 1 summarizes the structural characterization of a typical RF-sputtered LMO thin film on (100)-oriented SRO/STO. As shown schematically in Figure 1a, the sides and backside of the STO single-crystal substrate were sputter-coated with Ti/Pt to provide a good electrical contact between the subsequently deposited SRO thin-film current collector and working-electrode steel plunger of the test cell. The θ-2θ X-ray diffraction scan in Figure 1b clearly shows the (100), (200), and (300) reflexes of STO and SRO, with the SRO reflexes shifted to lower 2θ angles with respect to the substrate. More specifically, the (200) reflex is located at 46.49° and 45.19° for STO and SRO, respectively. In addition, the (400) LMO reflex is clearly visible at 43.97°. The corresponding out-of-plane lattice parameters amount to 3.904, 4.010, and 8.230 Å for STO, SRO, and LMO, respectively. For SRO, the elongated out-of-plane lattice parameter implies a significant compressive strain and tetragonal distortion. Reciprocal space mapping of the (103) SRO/STO reflex confirmed that the SRO thin film takes on the in-plane lattice parameter of the STO substrate, as shown in Figure S1 of the Supporting Information. For the LMO thin film, the out-of-plane parameter is virtually identical to that reported for bulk LMO. This, together with the absence of any additional LMO reflexes, suggests that LMO grows epitaxially on the SRO thin film with a significant compressive strain, but immediately relaxes to its bulk lattice parameter within a very short distance from the interface. Due to lack of a suitable reflex with sufficient signal intensity, reciprocal space mapping was not performed for the SRO/LMO films.

Figure 1d shows a high-resolution TEM image of the LMO/SRO interface as viewed along the [010] zone axis, confirming the heteroepitaxial growth of LMO on SRO. Despite the heavily strained interface, the film exhibits excellent crystallinity with a clear (200)_SRO_//(400)_LMO_ epitaxial relationship to the substrate, as shown schematically in Figure 1c. Given the large lattice mismatch of 5% between LMO and STO, the observed strain relaxation is hardly surprising. For the present analysis, the absence of strain is highly beneficial, as it means that the sample is representative of bulk LMO, and the extracted electrochemical properties can be understood as intrinsic material properties.

Figure 1e,f shows two AFM images of the thin-film surface at different magnifications. The sample exhibits the characteristic surface morphology of a (400)-oriented LMO film, which results from a preferential exposure of the ⟨111⟩ crystal facets.^17^ Statistical analysis of the AFM images revealed an RMS roughness of 30 nm and an effective surface area 21% higher than the nominal substrate area. The bright-field TEM image in Figure 1g is in good agreement with these AFM measurements, showing a dense thin film with pyramidal morphology characterized by well-defined angles, with an average thickness of approximately 80 nm. The maximum and minimum thicknesses of the film in the selected sample area were measured as roughly 150 and 30 nm, respectively. This strong variation of thickness leads to a continuous distribution of transport lengths throughout the sample and may cause some frequency dependency of the current distribution. However, owing to the absence of any significant porosity or tortuosity, we still consider the extraction of resistive or capacitive properties as meaningful. Furthermore, the consistent angles that characterize the film morphology should lead to a homogeneous thickness distribution between the extrema, meaning that the corresponding errors in the calculations of length-normalized properties (ionic conductivity and chemical capacitance) should be minor. For the following analysis, we therefore assume a flat thin film of 80 nm thickness.

For the calculations of the charge-transfer resistance, we normalize by the effective surface area measured by AFM, that is, 1.21 cm^2^. Current densities in the cyclic voltammetry (CV) scans are normalized by the substrate area (1 cm^2^). Although the XRD and TEM measurements indicate a dense, epitaxial thin film, the presence of some grain boundaries cannot be excluded, for example, between the pyramids. Assuming that such grain boundaries, if present, are poorly ion conducting as in other Li-ion conducting materials,^18^ this should not significantly impact the measured transport properties, and the extracted properties would still be close to those of the bulk material. Only if the grain boundaries allow fast ion conduction,^19^ the extracted properties would have to be regarded as effective rather than strictly bulk specific transport properties.

### DC Electrochemical Characterization

**Figure 2:**
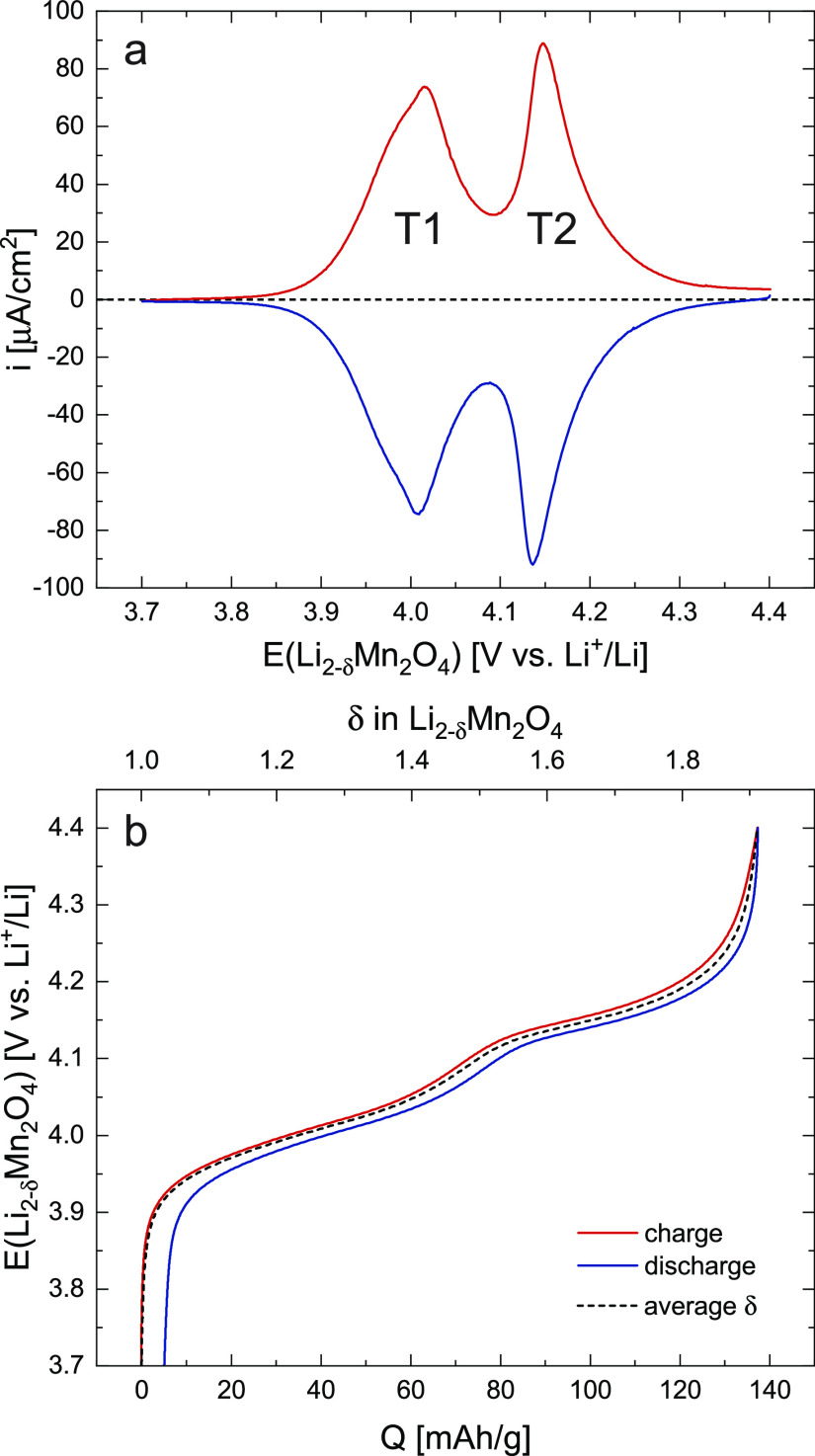
(a) Cyclic voltammogram (CV) of a fresh LMO thin-film electrode measured at a scan rate of 1 mV/s from 3.7 to 4.4 V. T1 and T2 denote the two nonequivalent types of tetrahedral sites due to Li ordering and mark the respective storage regimes. (b) Coulometric titration curves obtained by integration of the CV curves in panel a. The values of δ given at the top axis were obtained from the average charge at a given potential and shifted to δ = 1 at 3.7 V.

Figure 2a shows the CV curve of a typical LMO thin film measured versus Li metal at a scan rate of 1 mV/s from 3.7 to 4.4 V. The sample clearly exhibits two separate storage regimes, with two distinct CV peaks appearing at 4.01 and 4.14 V versus Li^+^/Li. These correspond to the emptying and filling of the previously described nonequivalent tetrahedral sites T1 and T2, which effectively differ in lattice site energy due to Li ordering at δ = 1.5. As expected from the different storage modes involved, the T1 peak is significantly broader (solid solution, FWHM = 111 mV) than the T2 peak (biphasic transition, FWHM = 65 mV). In agreement with literature,^3^ there appears to be an additional narrow peak superimposed on the T1 peak, suggesting the presence of a small miscibility gap within the T1 storage regime. Nonetheless, the solid-solution behavior remains dominant in this region, with little current added by the superimposed biphasic peak.

Despite the relatively high peak current densities of about 90 μA/cm^2^, kinetic overpotentials are small, with a charge/discharge hysteresis in the range of 10 mV, judging by the differences between the respective peak positions. The positive current offset at 4.4 V indicates a minor background current in the range of 1 μA/cm^2^ that decreases toward lower potentials and is even slightly negative (−0.5 μA/cm^2^) at 3.7 V. A charge/discharge curve, obtained by integration of the CV scan in Figure 2a, is shown in Figure 2b. Absolute values of charge were normalized by the thin-film mass, which was determined via the bulk density of LMO (4.29 g/cm^3^) by assuming a flat, single-crystalline thin film of 80 nm thickness. The LMO thin film exhibits a charge/discharge capacity of 137/132 mAh/g, corresponding to 93/89% of the theoretical capacity (148 mAh/g), in good agreement with commonly reported values,^6,20−22^ and a coulombic efficiency of 96%. The close agreement between the measured and theoretical capacities suggests a good electrical contact to the LMO film.

Background currents in DC measurements may play a much larger role for thin-film samples due to the low charge/discharge current densities in the μA/cm^2^ range, which could explain the relatively low coulombic efficiency compared to typical bulk electrode measurements. However, a second CV scan (not shown), measured after the extensive series of impedance measurements, was virtually identical to the initial scan. The coulombic inefficiency of 4% between charge and discharge can therefore safely be attributed to the observed background current in the CV scan, rather than any kind of material degradation.

Furthermore, the Li nonstoichiometry δ extracted from the charge/discharge curve as a function of electrode potential is indicated as a dashed line in Figure 2b, with the corresponding δ axis given at the top. Since the observed background current raises and lowers the effectively measured charge and discharge capacities, respectively, the reported values of δ were obtained from the average charge at a given potential and shifted to δ = 1 at 3.7 V. The resulting values are in the range of 1 ≤ δ ≤ 1.9, indicating a final charged state with a stoichiometry of Li_0.1_Mn_2_O_4_ at 4.4 V. The still incomplete extraction of Li at this cut-off voltage is in good agreement with previous reports.^3,12,23−26^

### Electrochemical Impedance Spectroscopy

**Figure 3:**
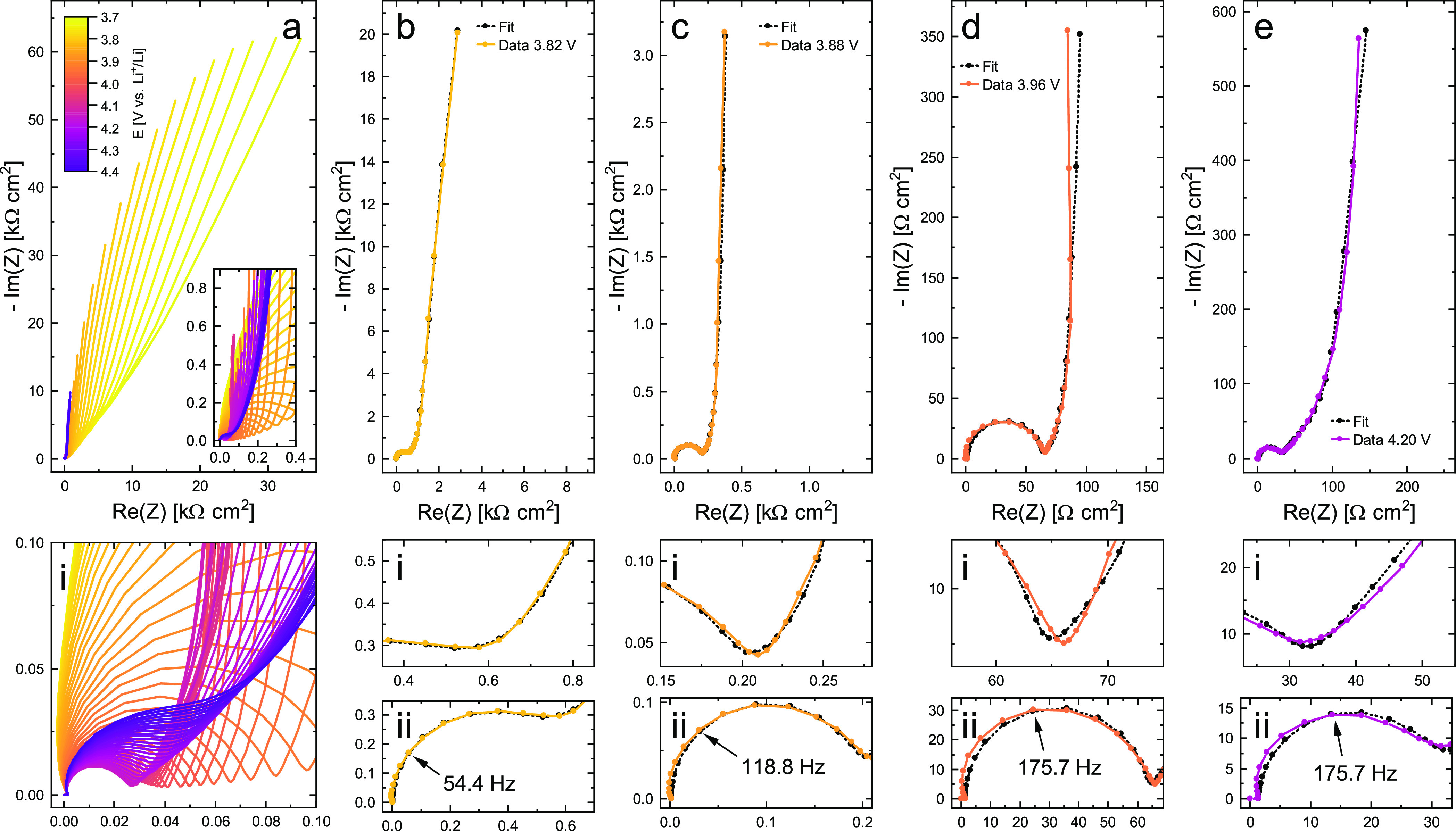
Series of impedance spectra at equilibrium electrode potentials of 3.7 to 4.4 V in intervals of 10 mV. Magnifications of the medium-to-high frequency regions are shown in subfigures (i) and (ii). (a) Full series of spectra, generally showing a significant decrease of real and imaginary impedance values from low to high electrode potential. The charge-transfer resistance reaches a minimum around 4.15 V. (b–e) Exemplary impedance spectra and fits at 3.82, 3.88, 3.96, and 4.20 V. The arrows in subfigures (ii) indicate the upper cut-off frequency used for the fit to exclude the slightly distorted high-frequency region.

Given the excellent reversibility of charge and discharge and the stability of the thin film, the electrochemical properties of LMO can be assumed to vary reversibly with electrode potential and hence Li activity, without any significant drift due to material degradation. Impedance spectra were measured for a broad range of stoichiometries to determine the charge-transfer resistance *R*_ct_, ionic conductivity σ_ion_, chemical capacitance *C*_chem_, and Li chemical diffusivity *D̃* as a function of SOC. A series of measurements, ranging from 3.7 to 4.4 V versus Li^+^/Li in potential increments of 10 mV, are shown as a Nyquist plot in Figure 3a and the corresponding magnification in Figure 3ai. Overall, the impedance spectra exhibit a strong dependence on electrode potential, with both real and imaginary parts varying over orders of magnitude. Starting at 3.7 V, both the real and imaginary parts of the spectra decrease with increasing potential, reaching a minimum around 4.0–4.1 V, and then increasing again toward 4.4 V. Qualitatively, this implies a maximum in chemical capacitance as well as minima in the interfacial and bulk transport resistances.

**Figure 4:**
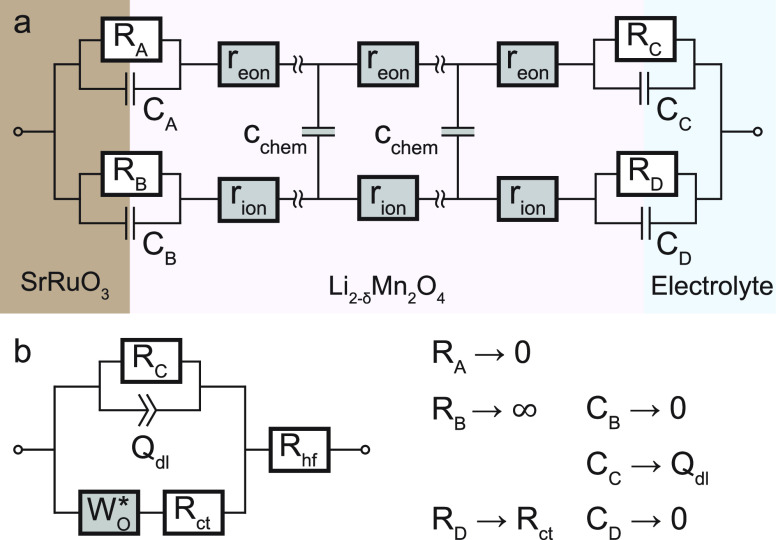
(a) General one-dimensional transmission line with four distinct terminals. The resistive and capacitive elements at the SRO/LMO and LMO/electrolyte interfaces can be adapted to account for selective blocking behavior. (b) Modified Randles’ circuit obtained by simplification of circuit (a) and replacement of the open Warburg element by an anomalous diffusion element *W*_O_^*^.

The general transmission line model (Figure 4a) first proposed by Jamnik and Maier^27−31^ describes the impedance of a one-dimensional current flow in a mixed conducting material such as an LMO electrode. This equivalent circuit consists of two resistive rails describing electronic and ionic transport (*R*_eon_ = ∑ *r*_eon_, *R*_ion_ = ∑ *r*_ion_) coupled by chemical capacitors (*C*_chem_ = ∑ *c*_chem_), with the chemical capacitance defined as^28,32^
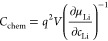
1where *q* is the elementary charge, *V* is the sample volume, μ_Li_ is the Li chemical potential, and *c*_Li_ is the concentration of formally neutral Li, that is, Li^+^ together with its electron. The Li chemical potential is defined by the fundamental relationship
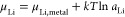
2with Boltzmann’s constant *k*, temperature *T*, the Li chemical potential of metallic Li μ_Li, metal_ (= reference potential) and the Li activity *a*_Li_. The Li activity is related to the electrode potential *E* versus Li metal via
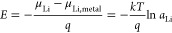
3

To simplify the equivalent circuit in Figure 4a for the case of an LMO electrode, we assume a high electronic conductivity such that *R*_eon_ = ∑ *r*_eon_ = 0. If the electronic conductivity was comparable to, or even lower than, the ionic conductivity, one would expect a notable SOC-dependent contribution to the high-frequency offset of the impedance spectra. This is not observed here, and we therefore consider the above assumption as reasonable. The chemical diffusion coefficient *D̃* can then be expressed as
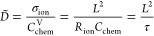
4where *C*_chem_^V^ is the volume-specific chemical capacitance, *L* is the film thickness, and τ = *R*_ion_*C*_chem_ is the time constant of the Li storage process.^33^ Furthermore, we assume that the SRO current collector presents an electronically ohmic contact and ionically blocking boundary to the mixed conductor and we neglect the corresponding interfacial capacitances, meaning that *R*_A_, *C*_B_ → 0 and *R*_B_ → ∞. At the LMO/electrolyte interface, we identify *C*_C_ and *R*_D_ as the double-layer capacitance *C*_dl_ and charge-transfer resistance *R*_ct_, respectively. Our experiments revealed that, particularly for small values of *C*_chem_^V^, side reactions may lead to some background current, that is, a finite DC resistance. This is considered in our impedance model by leaving a finite *R*_C_ in the circuit. However, the inclusion of *R*_C_ does not interfere with the further simplification of the circuit by replacing the transmission line with an open Warburg element *W*_o_. As in our previous work,^1^ we further replace the open Warburg element by an anomalous diffusion element *W*_o_^*^, implemented in the impedance-analyzing software EC-Lab (Biologic, France). This allows for a more general power-law dependence on the frequency, instead of the standard square-root behavior (ω)^1/2^. The corresponding impedance expression can be written as
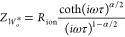
5with a nonideality parameter 0 ≤ α ≤ 1, and is similar to the impedance of the anomalous finite-space diffusion element reported by Bisquert.^34^ In the present study, α turned out to be in the range of 0.7–0.8.

Finally, we replace *C*_dl_ by a constant-phase element *Q*_dl_ and add a high-frequency offset resistance *R*_hf_ to account for the sum of resistances due to the electrolyte and other cell components to arrive at the equivalent circuit shown in Figure 4b. Please note that the placement of the double layer capacitance *C*_dl_ on the electronic (rather than the ionic) rail terminal is required to consider it in parallel to the open Warburg element. Only then, the model is consistent with the commonly used Randles’ circuit.^35,36^ In Figure S2 of the Supporting Information, the simulated impedance response of this circuit is shown in comparison to Randles’ circuit for a typical set of material parameters. Beside the change of the capacitive low-frequency end (Randles’ circuit) toward a large semicircle-onset (circuit c in Figure S2), the circuit in Figure 4b additionally features a steepening of the 45° regime and a flattening of the 90° regime, which adequately describes the empirical impedance spectra of most thin-film battery electrodes.^37−40^

**Figure 5:**
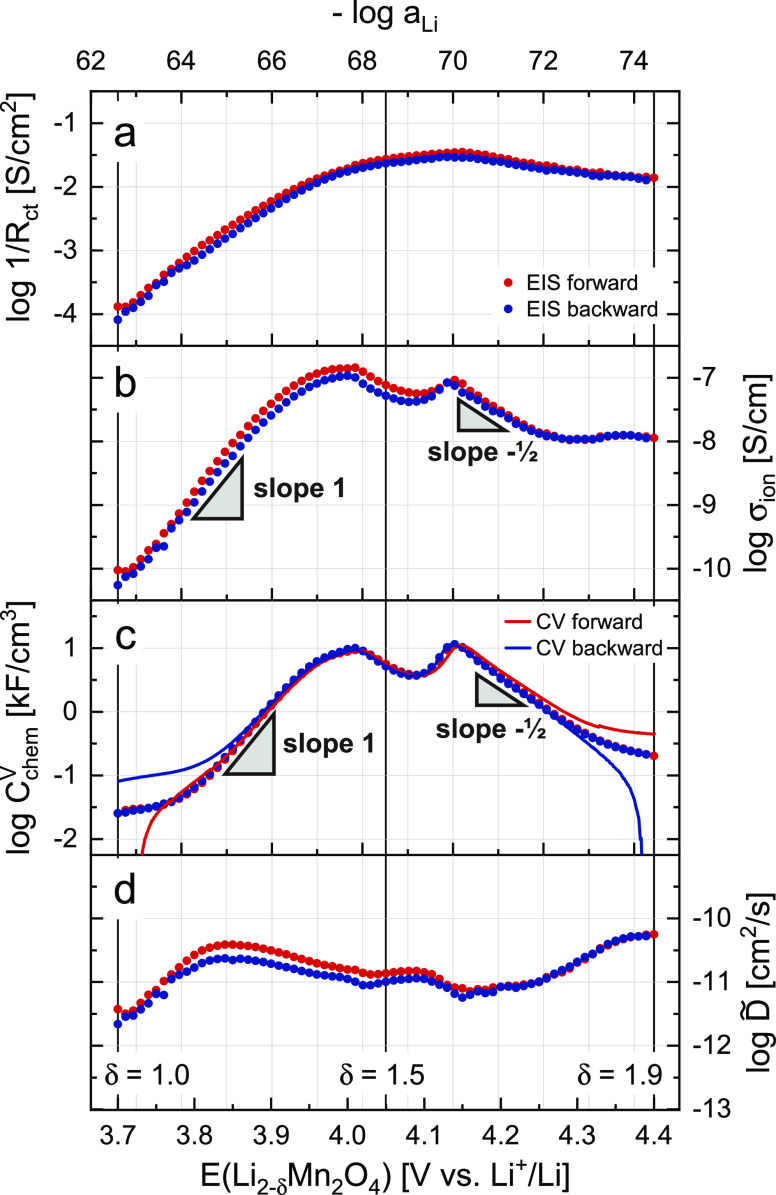
Electrochemical properties of the LMO thin film as a function of – log *a*_Li_ and electrode potential, as extracted from the impedance data. Results from the forward and backward scans are shown as red and blue dots, respectively. (a) Inverse charge-transfer resistance. (b) Ionic conductivity, slopes of 1 and −1/2 are indicated for the dilute regions. (c) Volume-specific chemical capacitance, values obtained from the CV scan are shown for comparison. (d) Chemical diffusion coefficient.

Figure 3b–e shows selected impedance spectra measured at 3.82, 3.88, 3.96, and 4.20 V with the corresponding fits. At 3.82 and 3.88 V, the quality of fit is excellent for almost the entire frequency range, with a minor deviation at the highest frequencies around the onset of the charge-transfer semicircle, where the spectra appear slightly distorted toward smaller real values. At 3.96 and 4.20 V, there is additionally a deviation in the low-frequency capacitive tail, with the spectra again appearing slightly distorted toward smaller real values at the lowest frequencies. We attribute these distortions to a minor geometrical misalignment of the square-shaped single-crystal substrate and Li counter electrode with respect to the concentric ring-shaped Li reference electrode. Nonetheless, the essential features of the impedance spectra are captured very well, and the extracted material properties vary continuously with electrode potential (see Figure 5). Furthermore, the validity of the extracted parameters is supported by their excellent agreement with DC measurements and thermodynamic theory, as demonstrated by the following analysis.

### Analysis of Material Parameters

Figure 5 shows the four essential material properties *R*_ct_, σ_ion_, *C*_chem_^V^, and *D̃* in a logarithmic plot as a function of electrode potential. The inverse of *R*_ct_ was plotted to emphasize the parallels and differences between the closely related properties *R*_ct_ and σ_ion_. Red and blue points indicate the values obtained from the impedance fits for the forward (3.70–4.40 V) and the backward (4.39–3.70 V) scan, respectively. The solid lines in Figure 5c additionally show the values of *C*_chem_^V^ obtained from the CV scan in Figure 2a via the relation
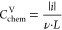
6with the current density *i* and the scan rate ν, where red and blue again indicate the forward and backward scan, respectively. On the top axis, – log *a*_Li_ is shown as calculated from the electrode potential via eq 3. All four properties show a strong dependence on the electrode potential, and hence on the SOC and Li activity, with *R*_ct_ and *C*_chem_^V^ varying over more than two, and σ_ion_ even changing over three orders of magnitude. The smallest variation is seen for *D̃*, with roughly one order of magnitude.

For the forward scan, starting at 3.70 V, *R*_ct_ decreases exponentially from an initial value of about 7600 Ω cm^2^ (1/*R*_ct_ ≈ 1.3 × 10^–4^ S/cm^2^) down to only 29 Ω cm^2^ at 4.15 V. Above 4.15 V, *R*_ct_ starts to increase again, reaching a nearly constant value of 70 Ω cm^2^ at 4.40 V. The obtained values are in good agreement with literature.^41,42^ In the backward scan, starting at 4.39 V, the values of *R*_ct_ are slightly higher, with a maximum deviation of about +29% around 3.85 V, but otherwise closely match those from the forward scan. Since *R*_ct_ does not only depend on the electrode’s surface concentration of ionic charge carriers, but furthermore varies with the concentration-dependence of the corresponding Galvani potential step across the LMO/electrolyte interface,^1,43^ its variation with Li activity can be highly complex and a mechanistic discussion is beyond the scope of this work. At this point, it is sufficient to state that the variation of *R*_ct_ qualitatively reflects the Li concentration in the material, transitioning from a vacancy-controlled insertion reaction with very few tetrahedral Li vacancies at low potentials to a Li^+^-controlled (high potential) insertion reaction. Accordingly, it reflects the two opposite defect regimes that will be further described in the defect chemical analysis.

The ionic conductivity σ_ion_, on the other hand, should be directly proportional to the concentration of the relevant ionic charge carriers, as long as the corresponding carrier mobilities remain relatively constant. Experimentally, σ_ion_ was found to vary over three orders of magnitude and roughly follows the double-peak shape of the CV curve in Figure 2a. As shown in Figure 5b, σ_ion_ increases exponentially from 10^–10^ S/cm at 3.70 V to more than 10^–7^ S/cm around 3.95–4.00 V. After a slight decrease and minimum around 4.08 V, σ_ion_ increases back to 10^–7^ S/cm at 4.14 V and subsequently starts to decrease exponentially until it reaches a near-constant value of 10^–8^ S/cm at 4.26 V and above.

Bulk ionic conductivity values of LMO have rarely been reported in the literature, and even fewer works describe its variation as a function of SOC. Guan and Liu obtained room-temperature ionic conductivities in the order of 10^–6^ S/cm by means of (nontrivial) electron blocking electrode impedance measurements on sintered pellets of nominally stoichiometric LiMn_2_O_4_ powder,^44^ which is four orders of magnitude higher than our values measured at the same stoichiometry. However, the high ionic conductivity measured in ref (44) might be due to insufficient equilibration times and low-end frequency range in the corresponding DC and impedance measurements, respectively. Although other explicit reports of ionic conductivities are hard to find, various thin-film studies report the SOC-dependent chemical diffusion coefficient together with differential capacities,^35,36,45−47^ from which the ionic conductivity can be roughly estimated (via eq 4) to be in the range of 10^–12^ to 10^–9^ S/cm, which is in good agreement with our data. Moreover, the strong variation of σ_ion_ with electrode potential at high and low SOC seen in Figure 5b is consistent with the strong variation of ionic charge carrier concentrations expected from the defect model (cf. next section) and we therefore consider our experimental values of σ_ion_ as meaningful. The two extended linear regions in Figure 5b around 3.70–3.90 and 4.14–4.26 V exhibit slopes β versus – log *a*_Li_ of about 1 and , respectively, and indicate regions where σ_ion_ with β = const. The absolute values of the observed slopes and their implication for the underlying defect chemical behavior will be discussed in the next section.

The volume-specific chemical capacitance *C*_chem_^V^, plotted in Figure 5c, qualitatively follows the same trend as σ_ion_ and, for the most part, is in excellent quantitative agreement with the values obtained from the CV scan in Figure 2a via eq 6. The additional capacitance seen in the CV data for the forward scan at high potentials and the backward scan at low potentials can be attributed to the previously described background currents in the cell. The diverging values in the forward and backward scan at low and high potentials, respectively, result from a reversal of the current direction in these regions following the reversal of the scan direction. The values of *C*_chem_^V^ obtained from the impedance fits are almost identical for the forward and backward scans, ranging from 25 F/cm^3^ at 3.70 V up to 9.3 kF/cm^3^ at 4.01 V and 11 kF/cm^3^ at 4.14 V, with a minimum of 3.8 kF/cm^3^ between the two maxima. From 4.14 V upward, *C*_chem_^V^ decreases again down to 200 F/cm^3^ at 4.40 V, with the corresponding slope in the log–log plot versus Li activity slightly flattening out above 4.30 V. Values of *C*_chem_^V^ have rarely been reported as volume-specific capacitances, but the corresponding CV current densities found in literature^41,42,48,49^ are similar to those shown in Figure 2a. As already seen for σ_ion_, the potential regions 3.80–3.95 and 4.14–4.30 V exhibit linear slopes of approximately 1 and , respectively.

**Figure 6:**
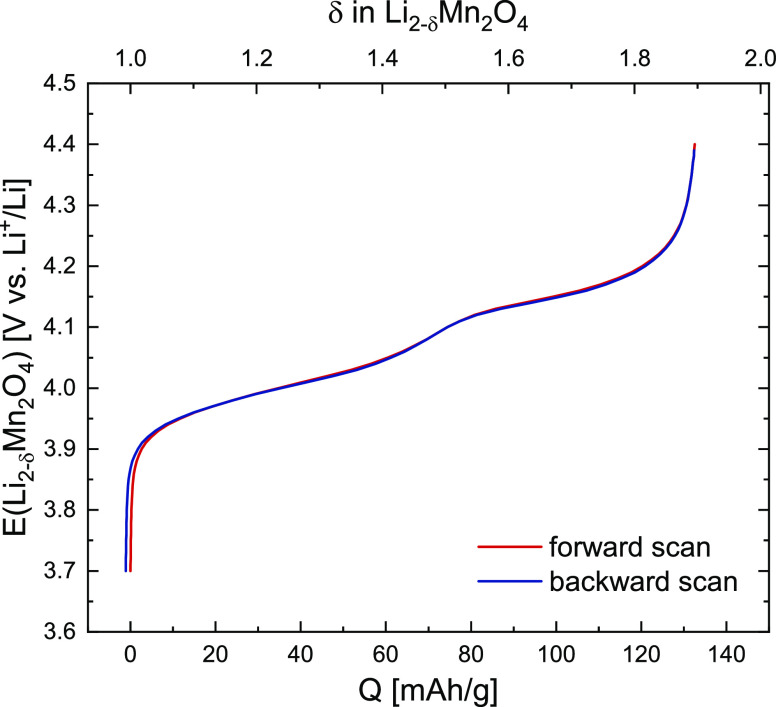
Equilibrium charge curve (OCV curve) obtained by integration of the chemical capacitance values from Figure 5c via eq 7. The values obtained for the forward (red) and backward (blue) scan are nearly identical. Both the charge and nonstoichiometry values agree very well with the CV data in Figure 2.

The chemical capacitance data from impedance measurements can also be integrated over the electrode potential, as shown in Figure 6, to obtain the total charge *Q* according to
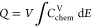
7The resulting charge/discharge curve should be unaltered by side reactions and truly reflects the relation between the equilibrium open-circuit potential and SOC. In fact, the impedance-based potential profile in Figure 6 is not only very similar to that obtained from CV measurements but also nearly identical for the forward and backward scan. This nicely demonstrates the fact that the chemical capacitance, and hence the equilibrium charge curve, is contained in the potential-dependent impedance response of a Li insertion electrode^41,50−52^ and, as an equilibrium property, can even be extracted more accurately from impedance spectra than from DC experiments. A direct comparison of the charge/discharge profiles obtained from CV and EIS is shown in Figure S3 of the Supporting Information.

Finally, the logarithmic chemical diffusivity can be calculated from σ_ion_ and *C*_chem_^V^ according to eq 4. It is shown in Figure 5d as a function of electrode potential and negative logarithmic Li activity. Starting at 3.70 V, *D̃* first increases from about 10^–11.5^ to 10^–10.5^ cm^2^/s at 3.85 V and then gradually decreases again down to 10^–11^ cm^2^/s at 4.25 V. Above 4.25 V, *D̃* increases again, up to a final value of about 10^–10.3^ cm^2^/s at 4.40 V. In agreement with eq 4, *D̃* remains rather constant in regions where σ_ion_ and *C*_chem_^V^ vary in concert (3.80–4.25 V). Overall, the obtained values of *D̃* are in good agreement with literature.^36,41,46,49,53^

### Defect Chemical Model

In terms of atomistic defect chemical considerations, the thermodynamically defined chemical capacitance *C*_chem_^V^ (cf. eq 1) is probably the most powerful material descriptor. It is often referred to as differential capacity or d*Q*/d*V* in the battery literature, and experimentally, it can be extracted from both AC impedance spectra and DC coulometric titration curves. To further evaluate *C*_chem_^V^ and μ_Li_ according to eqs 1 and 2, expressions for the dependence of all charged species on *a*_Li_ are required.^54^ In the following, these expressions will first be derived in generic form to describe Li insertion into (i) a material of the general formula Li_1–δ_MO_2_ with only one type of occupiable Li site, such as a layered oxide, and (ii) a material of the type Li_2–δ_M_2_O_4_ with two different Li sites, such as an ideal spinel that has octahedral and tetrahedral sites available for Li insertion. Finally, we will extend our defect chemical description to accurately describe the experimentally observed energetic splitting of tetrahedral sites in the specific case of Li_2–δ_Mn_2_O_4_ and compare the predicted values of *C*_chem_^V^ to the experimental data to validate our model.

We start by formulating the Li insertion equilibrium of Li_1–δ_MO_2_ in Kröger-Vink notation for the two relevant defects, that is,
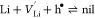
8Here, neutral Li must not be confused with Li ions in the cathode material. Rather, Li can be regarded as the combination of a Li ion in the electrolyte Li_yte_^+^ and an electron in the current collector e_cc_^–^ according to Li = Li_yte_^+^ + e_cc_^–^. The activity of this formally neutral Li is then defined by the voltage versus metallic Li (see eq 3), i.e., the electrochemical potential of the electrons in the current collecting phase, and thus also in the cathode. Only one type of Li site is available (*V*_Li_^′^ representing a vacant site) and the transition metal M is in the valence state of either +3 or + 4, with an electron hole h^•^ corresponding to M^4+^. The concentration [j] of each species (e.g., holes h^•^ and vacancies *V*_Li_^′^) can be referenced to the total concentration of formula units *c*^0^ according to
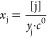
9where *x*_j_ is the site occupancy of species j and *y*_j_ is the number of the corresponding sites per formula unit. For example, *y*_h^•^_ = 1 and  = 1, since all Li sites are assumed to be filled for LiMO_2_. We neglect the interactions of all ionic and electronic charged species, as for dilute systems, but consider site restrictions that become relevant when broad variations of Li stoichiometry take place, that is, when a lattice site is almost completely emptied or filled.

The corresponding balance of chemical potentials reads
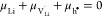
10with the individual site-restricted chemical potentials of vacancies and holes being^55,56^
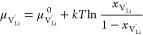
11and
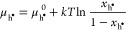
12where symbols μ_j_^0^ denote standard terms, that is, molar Gibbs free energies for noninteracting defects. Eqs 10, 11, and 12 can be combined to obtain the corresponding law of mass action
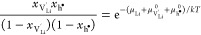
13In the absence of other charge carriers such as dopants, charge neutrality requires
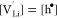
14and from eq 9, we thus obtain
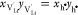
15For  = *y*_h^•^_ = 1, the concentrations of point defects (vacancies and holes) and their respective occupied sites are related via
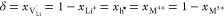
16where *x*_Li^+^_ indicates the fraction of Li sites occupied by Li^+^ and *x*_M^3+^_/*x*_M^4+^_ are the transition metal fractions in the respective valence states. From eq 13 and  = x_h^•^_, we obtain
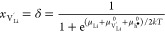
17and thus, the concentration of all four species is given as a function of Li chemical potential. In eq 17, μ_Li_ is related to the electrode potential and Li activity according to eq 3. For small defect concentrations, that is, before site restriction becomes relevant, eq 17 reduces to
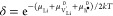
18According to eqs 1, 9, and 10, the chemical capacitance can be evaluated as
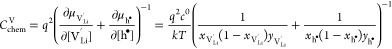
19which, for  = *x*_h^•^_ and  = *y*_h^•^_ = 1, becomes
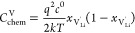
20

**Figure 7:**
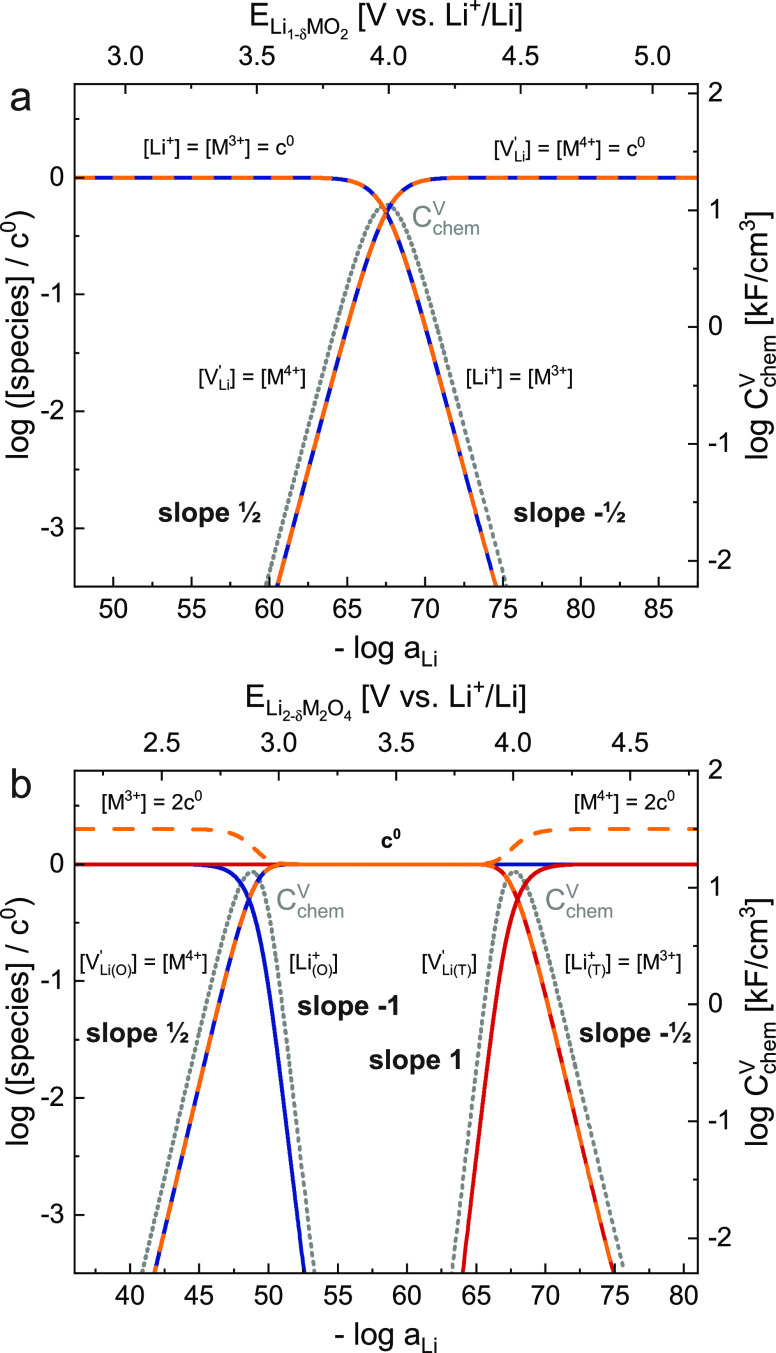
Calculated Brouwer diagrams for a generic (a) layered oxide Li_1–δ_MO_2_ (, μ_h^•^_^0^/*q* = 0.00 V,  = *y*_h^•^_ = 1) and (b) spinel Li_2–δ_M_2_O_4_ (, , μ_h^•^_^0^/*q* = 0.00 V, , *y*_h^•^_ = 2). The logarithmic site occupancies of all relevant species are plotted on the left, and the corresponding volume-specific chemical capacitance is plotted on the right *y*-axis as a function of – log *a*_Li_ (bottom) and electrode potential (top). For each occupiable Li lattice site, there is a corresponding peak in *C*_chem_^V^. For the spinel material, the difference of *y*_j_ for vacancies (1) and holes (2) leads to asymmetric Brouwer-slopes of the two *C*_chem_^V^ peaks. The volumes of one formula unit are assumed as 35 and 70 Å^3^ for Li_1–δ_MO_2_ and Li_2–δ_M_2_O_4_, respectively.

The concentrations of Li^+^, V_Li_^′^, M^3+^, and M^4+^ (h^•^) as calculated by eqs 16 and 17 and the corresponding chemical capacitance from eq 20 are plotted as a function of – log *a*_Li_ in Figure 7a for /*q* = 3.99 V and μ_h^•^_^0^/*q* = 0.00 V. Although the individual values of μ_*i*_^0^ are arbitrary in these generic model calculations, their sum determines the location of chemical capacitance peaks in the defect chemical model. The value of μ_h^•^_^0^ is arbitrarily set to zero, and  is then chosen such that ( + μ_h^•^_^0^)/*q* corresponds to the experimentally found *C*_chem_^V^ peak position of the first tetrahedral regime in Li_2–δ_Mn_2_O_4_ (see next section). For the sake of clarity, only V_Li_^′^ is written in Kröger-Vink notation. As expected from eq 18, [V_Li_^′^] and [M^4+^] both vary with a slope of  at high *a*_Li_ (low *E*), where [Li^+^] = [M^3+^] ≈ *c*^0^. Conversely, at low *a*_Li_ (high *E*), [Li^+^] and [M^3+^] vary with a slope of  while [V_Li_^′^] = [M^4+^] ≈ *c*^0^. The transition point between these two regimes marks the point of the highest chemical capacitance, where the concentration of all four species is the same. In this transition region, where the concentrations of vacant and occupied sites are similar in magnitude, site-limitations start to become relevant, and the point defect concentrations are accurately described by eq 17. The sum of  and μ_h^•^_^0^ defines the electrode potential where *C*_chem_^V^ peaks and the charge curve plateau is the flattest. The individual values of  and μ_h^•^_^0^, however, are chosen arbitrarily and are assumed to remain constant across the entire stoichiometry range.

It is worth mentioning that our chemical capacitance peak has a different reason than similar peaks found for acceptor-doped mixed conducting oxides used in high-temperature solid oxide fuel cells. There, the peak is caused by a change of the charge compensation mechanism from hole to oxygen vacancy compensation when changing the oxygen chemical potential.^57^ In our case, however, site restriction is key and it is always the smaller of the two concentrations  and 1 – , which dominates *C*_chem_^V^.

The above analysis can be extended for the case of two (or more) Li sites that share the same redox-active species. For each Li site *i*, an insertion equilibrium and the corresponding chemical potential balance can be formulated according to
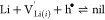
21
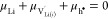
22Because all vacancies on available sites are in equilibrium with the same μ_Li_ and μ_h^•^_, it is immediately clear that the chemical potential of vacancies  must also be the same for all sites. Eqs 11 and 12 are still valid for μ_h^•^_ and each Li site individually. However, two laws of mass action result, one for each site (with different  and *y*_h^•^_ = 2), which are coupled by a more complicated charge neutrality equation. In principle, the balance of chemical potentials combined with the appropriate charge neutrality condition still defines the relevant point defect concentrations as a function of Li activity. However, the corresponding system of equations can no longer be solved analytically. This can be circumvented, at least partially, by expressing the total vacancy site fraction δ as
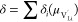
23Using eqs 9 and 11, we can express the dependence of the individual vacancy site fractions δ_*i*_ on the common vacancy potential  according to
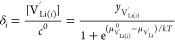
24For a hypothetical spinel cathode material Li_2–δ_M_2_O_4_ that offers octahedral (O) and tetrahedral (T) lattice sites for Li insertion,  and . The functional inverse of eq 23, , can be obtained numerically and inserted into eq 10 to arrive at the total Li chemical potential μ_Li_. The chemical potential of holes μ_h^•^_ is obtained directly from eq 12 with *y*_h^•^_ = 2 (*x*_h^•^_ = δ/2), since two M^3+^/M^4+^ redox centers are available per formula unit. For a better overview, the obtained chemical potential profiles of vacancies and holes are shown in Figure S4 of the Supporting Information, together with an explicit evaluation of eqs 23 and 24.

The resulting point defect concentrations (“Brouwer diagram”) are shown in Figure 7b in a log–log plot versus Li activity for μ_Li(O)_^0^/*q* = 2.90 V, , and μ_h^•^_^0^/*q* = 0.00 V. Due to the introduction of a second lattice site, the defect chemical behavior of Li_2–δ_M_2_O_4_ now exhibits four distinct subregimes, two for each site. Those shape the dependence of δ, and hence also *C*_chem_^V^, on *a*_Li_. Starting at a stoichiometry of Li_2_M_2_O_4_ (high *a*_Li_, low *E*), Li is extracted from the octahedral sites and [V_Li(O)_^′^] and [M^4+^] increase with a slope of , while [M^3+^] ≈ 2[Li_(O)_^+^] ≈ 2[Li_(T)_^+^] ≈ 2c^0^. Once most of the octahedral sites are empty, [Li_(O)_^+^] decreases with a slope of 1, while [M^3+^] ≈ [M^4+^] ≈ [V_Li(O)_^′^] ≈ [Li_(T)_^+^] ≈ *c*^0^. *C*_chem_^V^ reaches a maximum close to [V_Li(O)_^′^] = [Li_(O)_^+^] and the two decreasing sides of the *C*_chem_^V^ peak are again determined by the smaller of the two concentrations  and . The third subregime is entered when the electrode potential is further increased until [V_Li(T)_^′^] ≈ [Li_(O)_^+^], which occurs at a stoichiometry of LiM_2_O_4_. Li is now extracted from the tetrahedral sites and [V_Li(T)_^′^] increases with a slope of 1, together with *C*_chem_^V^. At the lowest activities and highest potentials, once most of the tetrahedral sites are empty, [Li_(T)_^+^] and [M^3+^] decrease with a slope of , while [M^4+^] ≈ 2[V_Li(O)_^′^] ≈ 2[V_Li(T)_^′^] ≈ 2*c*^0^. In the transition region between these last two subregimes, *C*_chem_^V^ exhibits another peak close to [V_Li(T)_^′^] = [Li_(T)_^+^]. It is therefore evident that, for every lattice site available for Li insertion and extraction, there is an associated peak in *C*_chem_^V^.

### Application to the Chemical Capacitance of Li_2–δ_Mn_2_O_4_

Finally, this defect chemical description can be adapted to correctly describe the reported experimental behavior of a Li_2–δ_Mn_2_O_4_ electrode. The observed Li ordering close to δ = 1.5 leads to an energetic splitting of the tetrahedral 8a sites, which is taken into account by assuming two different tetrahedral sites in the entire 1 ≤ δ ≤ 2 range, labeled T1 and T2, with . This leads to two separate peaks in *C*_chem_^V^ without any stepwise changes. Conversely, if fully equivalent tetrahedral sites were assumed with a sudden split into T1 and T2 at δ = 1.5, *C*_chem_^V^ would be expected to show a sudden step, contrarily to what is observed experimentally. Furthermore, we exclude all two-phase regions from our analysis, since the defect chemical description above relies on the presence of a solid solution. The Li chemical potential, chemical capacitance, and point defect concentrations are obtained analogously to the previously described general case of Li_2–δ_M_2_O_4_, with a third Li site introduced due to the tetrahedral site splitting. The corresponding sites are referred to as O, T1, and T2 with sites per formula unit  and . The standard potentials μ_*i*_^0^ are chosen such that the *C*_chem_^V^ peaks appear at the same electrode potentials as observed experimentally for Li_2–δ_Mn_2_O_4_, that is, , μ_Li(T1)_^0^/*q* = 3.99 V, , and μ_h^•^_^0^/*q* = 0.00 V. Please note that these standard values differ slightly from the *C*_chem_^V^ peak positions due to the additional concentration-dependent contribution of μ_h^•^_ to the total Li chemical potential. This is further illustrated in Figure S4 of the Supporting Information.

**Figure 8:**
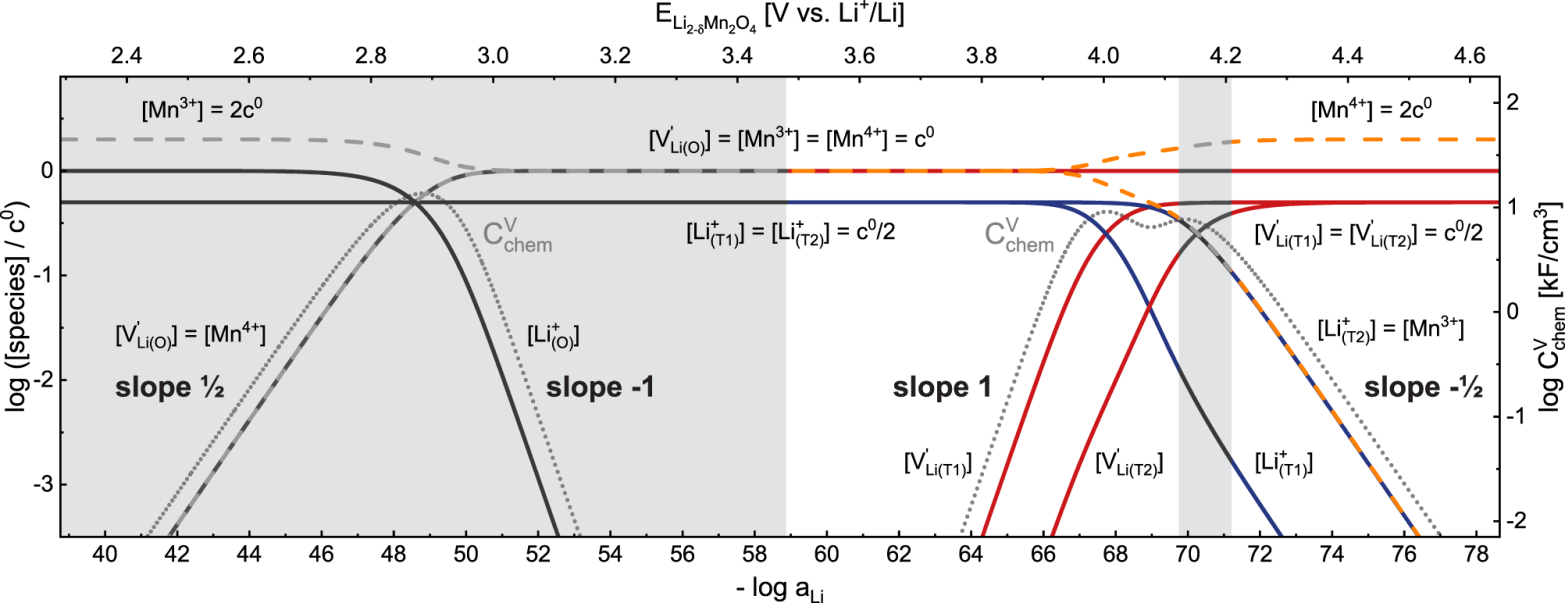
Calculated Brouwer diagram for Li_2–δ_Mn_2_O_4_ (, , , μ_h^•^_^0^/*q* = 0.00 V, , ). The logarithmic site occupancies of all relevant species are plotted on the left, and the corresponding volume-specific chemical capacitance is plotted on the right *y*-axis as a function of – log a_Li_ (bottom) and electrode potential (top). Approximate regions of reported two-phase regimes (0 ≤ δ ≤ 1 and 1.65 ≤ δ ≤ 1.9) of Li_2–δ_Mn_2_O_4_ are grayed-out, since the defect chemical model relies on the presence of a single-phase solid solution.

The resulting full Brouwer diagram of Li_2–δ_M_2_O_4_ is shown in Figure 8, where regions are marked in gray that are known experimentally to behave as two-phase regimes rather than solid solutions for M = Mn. The storage regime involving tetrahedral sites, around – log *a*_Li_ ≈ 68 (*E* ≈ 4 V versus Li), is now split into two separate regimes with a corresponding double peak in *C*_chem_^V^, that is characterized by slopes of 1 and −1/2 at high and low Li activities, respectively. The two peaks differ in their shape and absolute values due to the asymmetric behavior of the electronic charge carriers for 1 ≤ δ ≤ 2.

**Figure 9:**
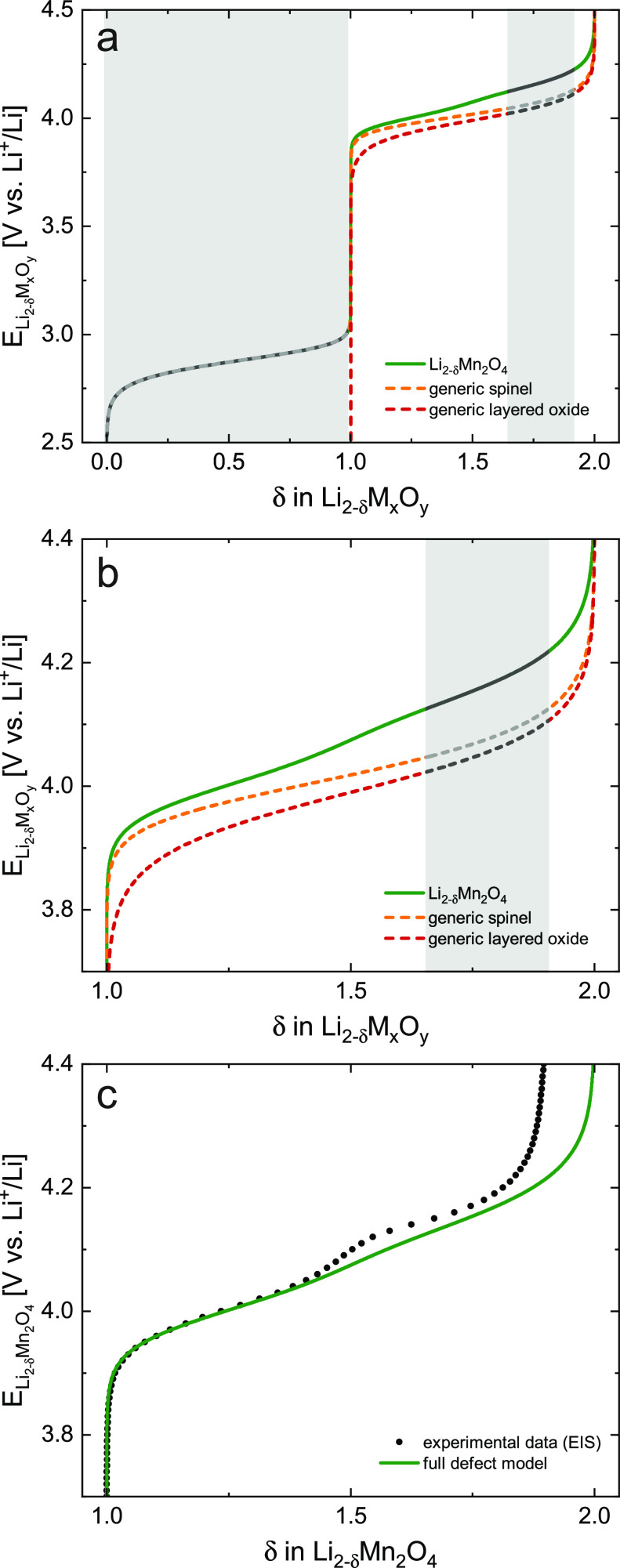
(a, b) Calculated electrode potential profiles as a function of δ for (i) a generic layered oxide (red), (ii) a generic spinel (orange), and (iii) Li_2–δ_Mn_2_O_4_ (green), corresponding to the defect models presented in Figure 7a, Figure 7b, and Figure 8, respectively. Approximate regions of reported two-phase regimes (0 ≤ δ ≤ 1 and 1.65 ≤ δ ≤ 1.9) of Li_2–δ_Mn_2_O_4_ are grayed-out, since the defect chemical model relies on the presence of a single-phase solid solution. (a) Full charge curve for 0 ≤ δ ≤ 2. (b) Magnification of the tetrahedral-site regime with nonstoichiometries 1 ≤ δ ≤ 2. (c) Comparison of the calculated charge curve (full defect model) with the experimental data from EIS, both being in good agreement in the low-voltage region. In the mid- and high-voltage regions, the experimental data deviate from the defect calculations due to the presence of a two-phase regime and incomplete Li extraction.

To arrive at a more conventional representation of the presented defect model, the calculated chemical potential μ_Li_ can also be converted into a charge curve, that is, an electrode potential versus Li as a function of nonstoichiometry δ, via eq 3. The resulting charge curves for the three presented cases of (i) generic layered oxide, (ii) generic spinel, and (iii) Li_2–δ_Mn_2_O_4_ (without two-phase regions) are shown in Figure 9a,b. The generic spinel differs from the generic layered oxide in two essential aspects. First, the spinel structure allows the insertion of a second formula unit of Li by occupying the vacant octahedral sites at a lower electrode potential. Second, due to the availability of two redox active transition metals per formula unit, compared to only one per formula unit for each type of lattice site, the concentration of electronic charge carriers is very high and nearly constant on a logarithmic scale in the region around 3.5 V, where the concentrations of Li_(O)_^+^ and V_Li(T)_^′^ are very small and vary over orders of magnitude (cf. Figures 7 and 8). As a result, the logarithmic increase in electrode potential upon removal of Li from the tetrahedral sites is only limited by the concentration of tetrahedral vacancies and the charge curve plateau is therefore flatter than for the layered oxide, as shown in Figure 9b.

Finally, the charge curve of Li_2–δ_Mn_2_O_4_ differs from the generic spinel due to Li ordering at δ = 1.5, which leads to a splitting of the tetrahedral site plateau into the characteristic double plateau around 4 V. The resulting Li_2–δ_Mn_2_O_4_ charge curve exhibits steeper plateau regions than the generic spinel, more similar to the generic layered oxide, with a small potential step around δ = 1.5. Please note that ionic ordering is not strictly specific to spinel cathode materials - Li ordering at half occupancy has also been reported, for example, for layered Li_0.5_CoO_2_, where it also causes a visible potential step in the charge curve.^58−60^

**Figure 10:**
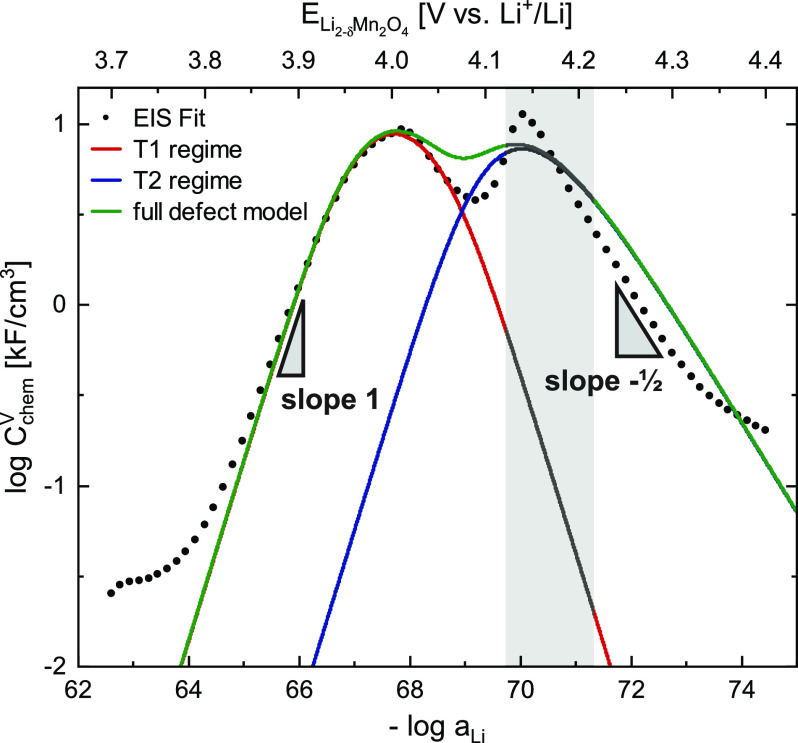
Calculated and measured (EIS) volume-specific chemical capacitance as a function of – log *a*_Li_ and electrode potential. The approximate region of the reported two-phase regime (1.65 ≤ δ ≤ 1.9) of Li_2–δ_Mn_2_O_4_ is grayed-out, since the defect chemical model relies on the presence of a single-phase solid solution. The green line represents the values of *C*_chem_^V^ calculated from the full impedance model (Figure 8), while the red and blue lines represent the calculated values from the isolated T1 and T2 regimes, respectively.

To verify the proposed defect model for Li_2–δ_Mn_2_O_4_, the predicted values of *C*_chem_^V^ (green continuous line) are plotted in Figure 10 together with those obtained from impedance measurements (dots). For a more detailed analysis, the calculated chemical capacitances of the isolated T1 and T2 regimes are plotted in red and blue, respectively, with contributions of all other lattice sites to the total chemical potential of vacancies μ_V_Li_^′^_ being neglected. In the T1 regime, the values of *C*_chem_^V^ predicted by the defect model are in excellent qualitative and quantitative agreement, in terms of both absolute values and slopes. In the T2 regime, the general shape of the experimental data is also correctly reproduced, especially the slope of  at high potentials. However, as expected due to the presence of a two-phase regime in this potential region, the experimental data exhibit a sharper peak in *C*_chem_^V^ than predicted by the defect chemical single-phase model. Since such a phase transition implies that a certain fraction of the electrode capacity is filled or emptied at a fixed electrode potential, this can also explain why the experimentally observed decrease with a slope of  is shifted to lower potentials with respect to the model calculations.

The experimental data can also be compared to the charge curve calculated from the proposed Li_2–δ_Mn_2_O_4_ defect model, as shown in Figure 9c. At low values of δ, up to δ ≈ 1.4, both curves are in good agreement. Above δ = 1.4, the experimental data deviate from the calculated curve due to the two-phase regime. After the two-phase regime, above δ ≈ 1.8, the experimental charge curve slopes upward to reach a maximum degree of Li extraction of δ = 1.9 at 4.4 V, deviating from the theoretical maximum of δ = 2.0 due to the previously described incomplete Li extraction. Nonetheless, the similar shape of the experimental and calculated charge curves in the high-voltage region suggests that the defect model could in principle also describe the voltage profile of Li_2–δ_Mn_2_O_4_ for δ > 1.8, if appropriate corrections for incomplete Li extraction and the two-phase regime were introduced.

The good agreement of our dilute defect model with the experimental data over a rather wide stoichiometry range is somewhat surprising, given the high carrier concentrations involved. In a similar electrochemical study on Li_1-δ_CoO_2_, substantial deviations from the simple model without a defect interaction already appeared at about 10% Li vacancies.^1^ In general, defect interactions (or other changes of the materials with varying defect concentrations) seem to be less relevant for the spinel-type electrode compared to layered oxides; this is already visible in the steeper slopes and irregularities of the plateau regions for layered cathodes. Exact reasons for these differences can be manifold and may include the anisotropic volume changes of layered oxides upon cycling, which makes it usually hard to distinguish between ionic defect interactions and interactions with the gradually changing host lattice. Nonetheless, nonidealities due to defect interactions are probably also present in spinel-type materials and might, for example, cause the mismatch between the calculated chemical capacitance minimum around 4.08 V (green curve in Figure 10) and the measured minimum.

### Analysis of the Ionic Conductivity of Li_2–δ_Mn_2_O_4_

The shape of the potential-dependent ionic conductivity curve (Figure 5b) strongly resembles that of *C*_chem_^V^, with the characteristic double peak and slopes of 1 and , for low and high potentials, respectively. This can again be understood from the defect concentrations. The transport of Li^+^ throughout the tetrahedral sublattice takes place via octahedral sites^61−63^ and, phenomenologically, can be viewed as a second order reaction between Li^+^ and a tetrahedral Li vacancy. For independent motion on the T1 and T2 sublattices, the ionic conductivity σ_ion_ can thus be approximated by
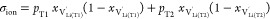
25The prefactors *p*_T1_ and *p*_T2_ are site-specific proportionality factors and resemble the mobility factors when writing the ionic conductivity in terms of one defect concentration only. For each sublattice, this corresponds to a transition from vacancy-limited (, only few vacancies) to Li^+^-limited (, only few ions on the relevant sites) ion conduction, analogously to *C*_chem_^V^ in eq 20. For constant prefactors *p_i_* and assuming only jumps within a given sublattice (T1, T2), the total ionic conductivity versus – log *a*_Li_ curve predicted by eq 25 therefore shows the same general shape as *C*_chem_^V^ in Figure 10, in accordance with the experimental data (Figure 5b). However, while σ_ion_ in eq 25 only depends on ionic site fractions, the general *C*_chem_^V^ term also includes *x*_h^•^_ contributions and this may cause some quantitative deviations. Further differences between the σ_ion_ and C_chem_^V^ may be attributed to the absence of any defect interaction in our model, since the interaction supposedly affects thermodynamics (concentrations) as well as kinetics (*p_i_*-factors).

Given the ionic conductivities in Figure 5b and the ionic charge carrier concentrations in Figure 8, an effective ionic carrier mobility *u*_*i*,eff_ can be obtained based on the fundamental relationship
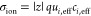
26where *z* = 1 is the charge number and *c*_*i*,eff_ is a kind of effective concentration of ionic charge carriers on site *i* according to
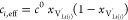
27with site concentration *c*^0^. Assuming only T1 sites contributing to the ionic conductivity for 1 ≤ δ ≤ 1.5 and only T2 sites for 1.5 ≤ δ ≤ 1.9, we can separate eq 25 into its T1 and T2 terms and arrive at expressions for *u*_T1,eff_ (1 ≤ δ ≤ 1.5) and *u*_T2,eff_(1.5 ≤ δ ≤ 1.9) according to
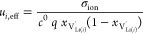
28

**Figure 11:**
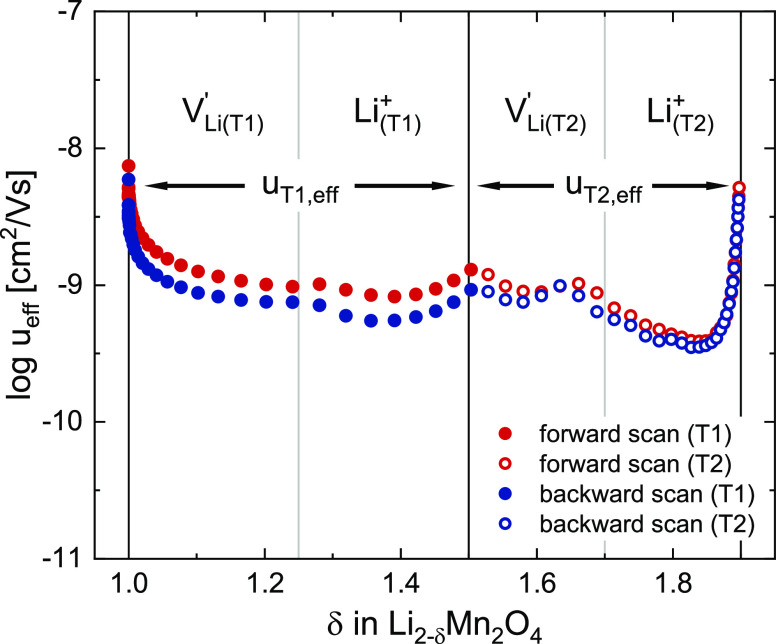
Effective ionic mobilities as a function of δ. For the T1 (1 ≤ δ ≤ 1.5) and T2 (1.5 ≤ δ ≤ 1.9) regions, values of *u*_*i*,eff_ were calculated via eq 28 by inserting the corresponding vacancy site fractions  from Figure 8.

The effective site mobilities are plotted in Figure 11, where the δ regions of the T1 and T2 regimes are indicated together with the respective limiting ionic charge carriers. The capacity of the tetrahedral regime was scaled down to 1.0 ≤ δ ≤ 1.9 to correct for the experimentally observed incomplete Li extraction. Starting at δ = 1, the effective ionic mobility initially drops down from approximately 10^–8^ to 10^–9^ cm^2^/Vs and then remains relatively constant over most of the compositional range. Close to δ = 1.9, the mobility increases again from 10^–9.5^ to 10^–8.2^ cm^2^/Vs. The initial rather sharp drop close to δ = 1 reflects the slope of log σ_ion_ being much lower than the slope of 1 predicted by the defect model for log [V_Li(T1)_^′^] at electrode potentials close to 3.7 V (see Figure 5). The sharp increase close to δ = 1.9 is due to σ_ion_ remaining nearly constant above 4.3 V, where the defect model predicts a slope of  for log [Li_(T2)_^+^]. This nominal increase in mobility can be considered an artifact, since the main contribution to σ_ion_ in this potential region presumably comes from the remaining Li^+^ (incomplete extraction) in the material, which is only removed at potentials above 4.4 V and is not considered in our model. We therefore consider the ionic mobility of both sites (and thus also the limiting mobilities of the four ionic charge carriers) to be close to 10^–9^ cm^2^/Vs for the investigated stoichiometry range.

Finally, we may briefly consider the Li chemical diffusion coefficient in Figure 5d, determined from the σ_ion_ and *C*_chem_^V^ according to eq 4. In the simplest case of a generic layered oxide (eq 20 and ), we find even analytically a constant value of *D̃*. Some variations come into play due to different *p_i_*-factors for different sites, concentration-dependent *p_i_*, *x*_h^•^_-terms in *C*_chem_^V^, and the consequences of the defect interactions mentioned above. A more detailed discussion of the rather modest *D̃* changes, however, is beyond the scope of this paper.

## Conclusions

Epitaxial thin films of spinel-type Li_2–δ_Mn_2_O_4_ were sputter-deposited on (100)-oriented SrRuO_3_/SrTiO_3_ substrates and analyzed electrochemically by means of cyclic voltammetry and impedance spectroscopy. The thin-film electrodes exhibited excellent electrochemical reversibility, thus allowing the reliable extraction of a complete set of electrochemical properties from impedance measurements as a function of SOC for a broad potential range of 3.70–4.40 V versus Li. These properties consist of the charge-transfer resistance *R*_ct_, ionic conductivity σ_ion_, volume-specific chemical capacitance *C*_chem_^V^, and chemical diffusivity *D̃*. The equilibrium open-circuit potential profile could be accurately reconstructed via integration of the *C*_chem_^V^ data from impedance fits, highlighting the central role of the chemical capacitance as a fundamental thermodynamic property of Li insertion materials.

A defect chemical model was deduced, which describes the charge (δ) dependence of the electrode potential of Li_2–δ_Mn_2_O_4_ versus Li as the combination of a single-site-restricted electron hole potential μ_h^•^_ and multisite-restricted Li vacancy potential . The model is in excellent qualitative and quantitative agreement with the experimentally obtained values of *C*_chem_^V^. Characteristic peaks of the chemical capacitance always occur around half occupancy of a certain crystallographic site. A double peak is introduced in Li_2–δ_Mn_2_O_4_ by the splitting of tetrahedral sites into two types of sites (vacancy ordering). Significant deviations from the model were only observed in the potential region around 4.1–4.2 V, where a phase separation is known to occur. These results demonstrate that the chemical potential and associated electrochemical properties of a solid-solution Li insertion material can be rather accurately described by simple concentration dependences of the individual point defect chemical potentials of ionic and electronic charge carriers, when taking account of the lattice site restrictions imposed by the material’s crystal structure. The presented model can easily be adapted for different transition metal stoichiometries and doping states, and therefore opens the gates toward a better defect chemical understanding of the entire class of spinel cathode materials.

## Supporting Information Available

Reciprocal space map of SRO/STO interface, detailed derivation and simulations of impedance model, comparison of AC and DC charge curves, and explicit comparison of μ_Li_, μ_h^•^_, and  (PDF)
