# Supplementary material for: Defect Chemistry of Spinel Cathode Materials—A Case Study of Epitaxial LiMn2O4 Thin Films
Source: Chem Mater. 2023 Jun 29;35(13):5135–49. doi: 10.1021/acs.chemmater.3c00814 (PMC10339684; doi:10.1021/acs.chemmater.3c00814)
Supplement: Supplementary file 1 — cm3c00814_si_001.pdf [file cm3c00814_si_001.pdf]

Supporting Information

Defect Chemistry of Spinel Cathode Materials – A Case Study of Epitaxial $LiMn_2O_4$ Thin Films

Andreas E. Bumberger,[a*] Christin Böhme [a], Joseph Ring [a], Sergej Raznjevic [b], Zaoli Zhang [b] , Markus Kubicek [a] and Juergen Fleig [a]

[a] *Institute of Chemical Technologies and Analytics, TU Wien, Vienna, Austria*

[b] *Erich Schmid Institute for Materials Science, Leoben, Austria*

* corresponding author, andreas.bumberger@tuwien.ac.at

Reciprocal Space Mapping

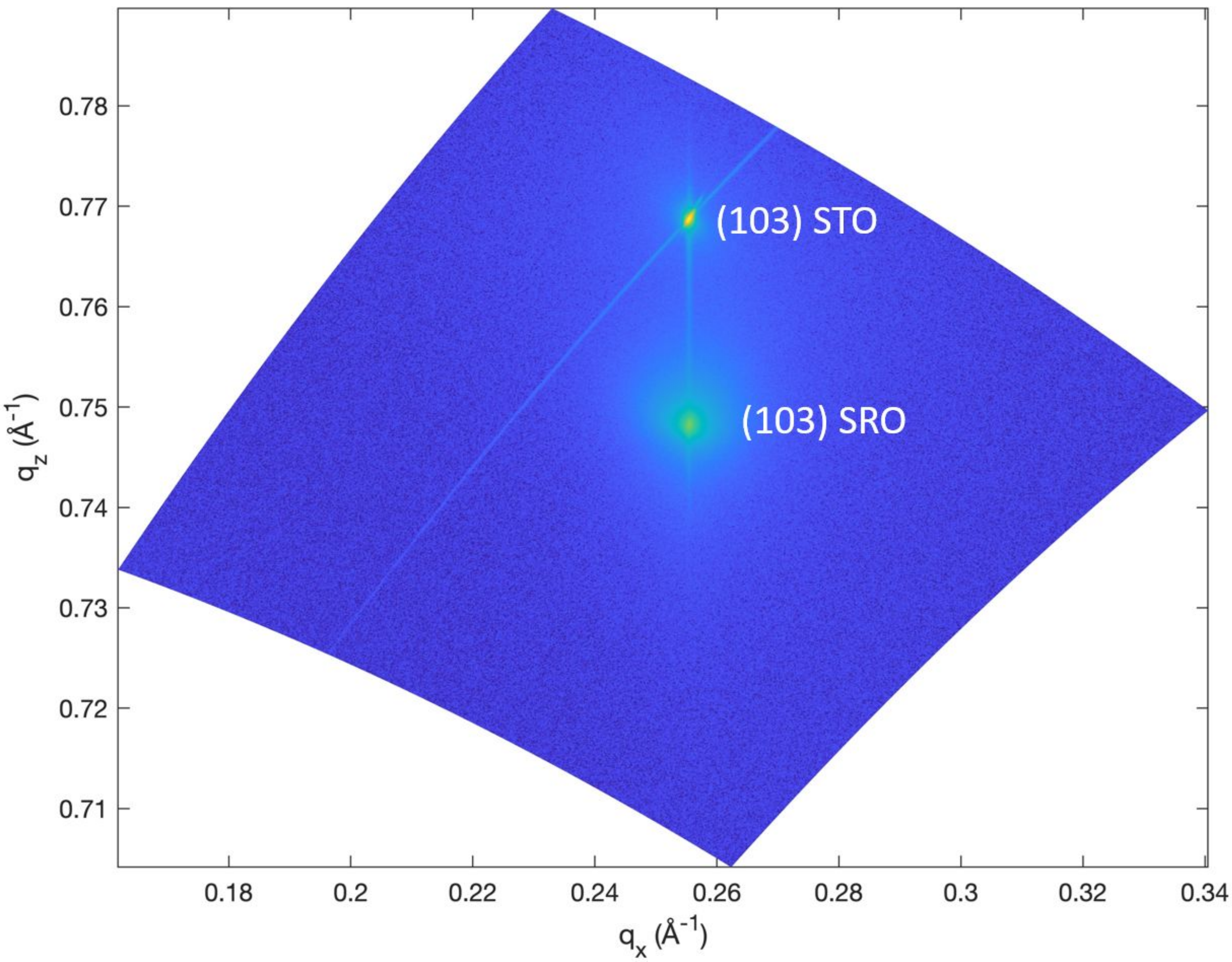

Figure S1. Reciprocal space map of the (103) STO/SRO reflex of the sample shown in Figure 1 of the main manuscript. The alignment of the q_x vectors of SRO and STO indicates that both films share the same in-plane lattice parameter.

Impedance model and simulated spectra

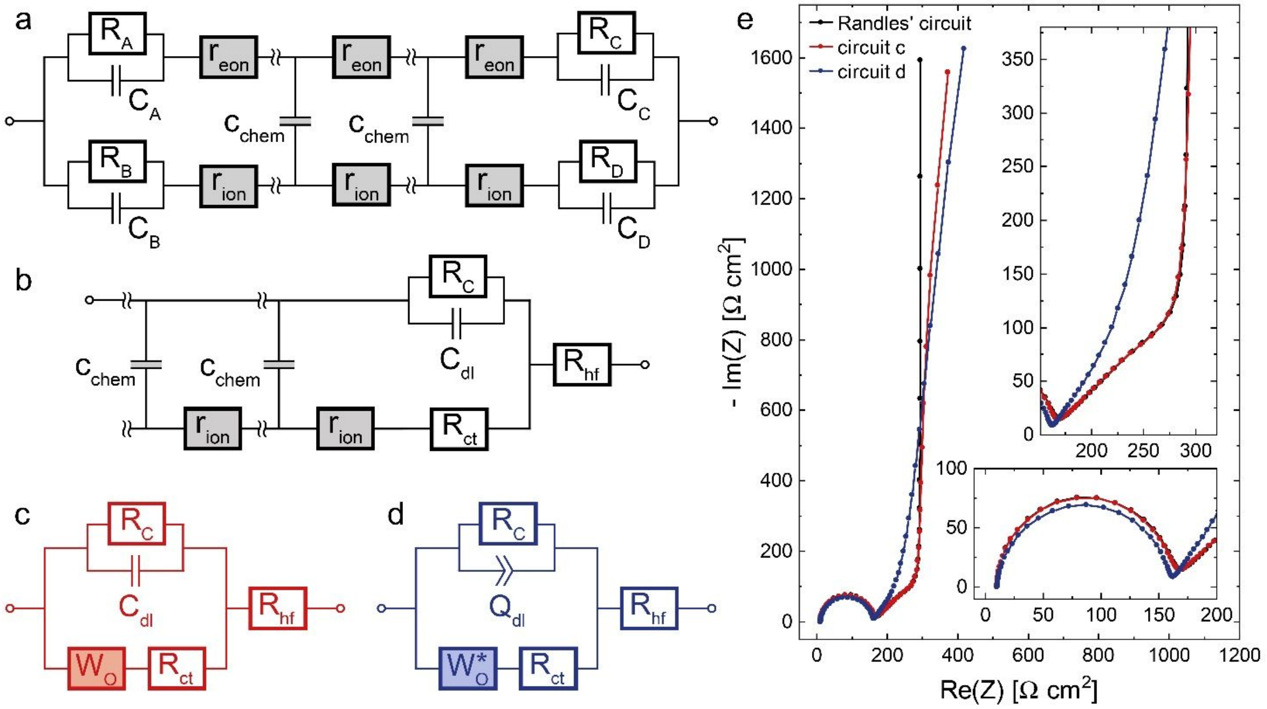

Figure S2 (a) Full one-dimensional transmission line with four distinct interfacial terminals. (b) Simplified transmission line model representing a thin-film LMO electrode, taking into account a finite resistance R_C due to side reactions. (c) Simplified representation of circuit (b) obtained by replacing the remaining transmission line by an open Warburg element W_O. The resulting circuit is a modified Randles' circuit, with an additional resistance R_C. (d) Equivalent circuit used for the fitting of impedance spectra in this work. The open Warburg element was replaced by an anomalous diffusion element W_O^*, with an impedance response given by equation S1. (e) Simulated impedance response of Randles' circuit ($R_{hf} = 10\ \Omega$, $R_{ct} = 150\ \Omega$, $C_{dl} = 10\ \mu F$, $R_{ion} = 400\ \Omega$, $C_{chem} = 100\ mF$) compared to circuits (c) and (d). For circuit (c) a resistance $R_C = 300\ k\Omega$ was used. For circuit (d) a constant-phase exponent of 0.95 with $Q_{dl} = 10\ \mu F\ s^{-0.05}$ was used for the constant-phase element, together with a nonideality factor $\alpha = 0.75$ for the anomalous diffusion element.

$$Z_{W_o^*} = R_{ion} \frac{\coth{(i\omega\tau)^{\alpha/2}}}{(i\omega\tau)^{1-\alpha/2}} \tag{S1}$$

Comparison of Charge Curves from Cyclic Voltammetry and Impedance Spectroscopy

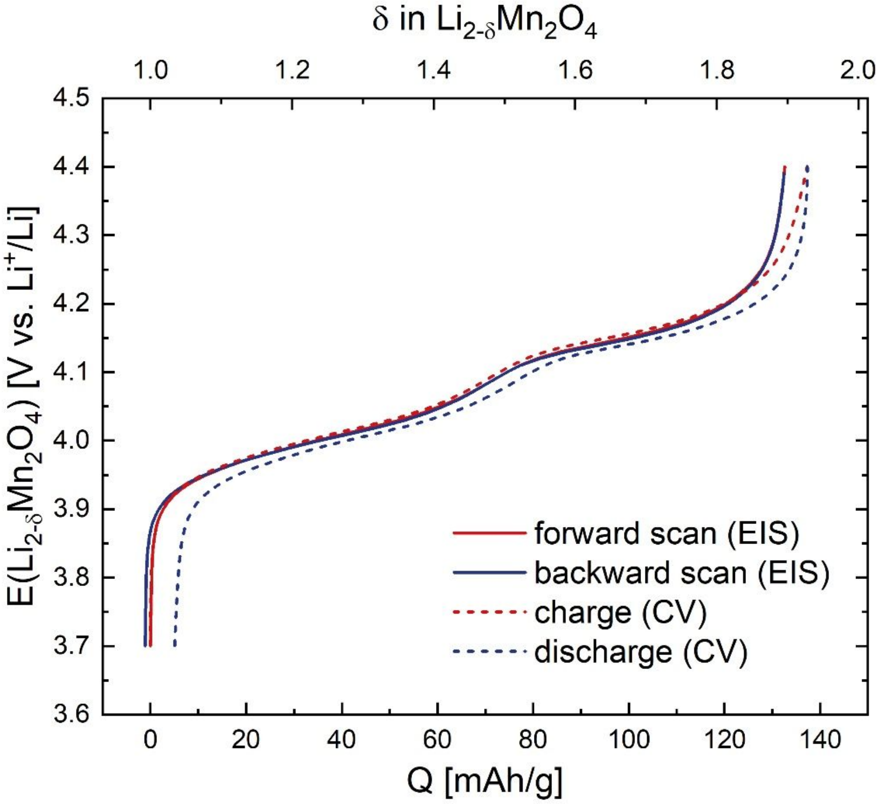

Figure S3. Comparison of the charge curves obtained via electrochemical impedance spectroscopy (EIS) and cyclic voltammetry (CV). The CV data show a higher charge capacity and lower coulombic efficiency due to background currents and a slight voltage hysteresis due to overpotentials. The charge/discharge curves from EIS show minimal discrepancy.

Multi-site-restricted chemical potential

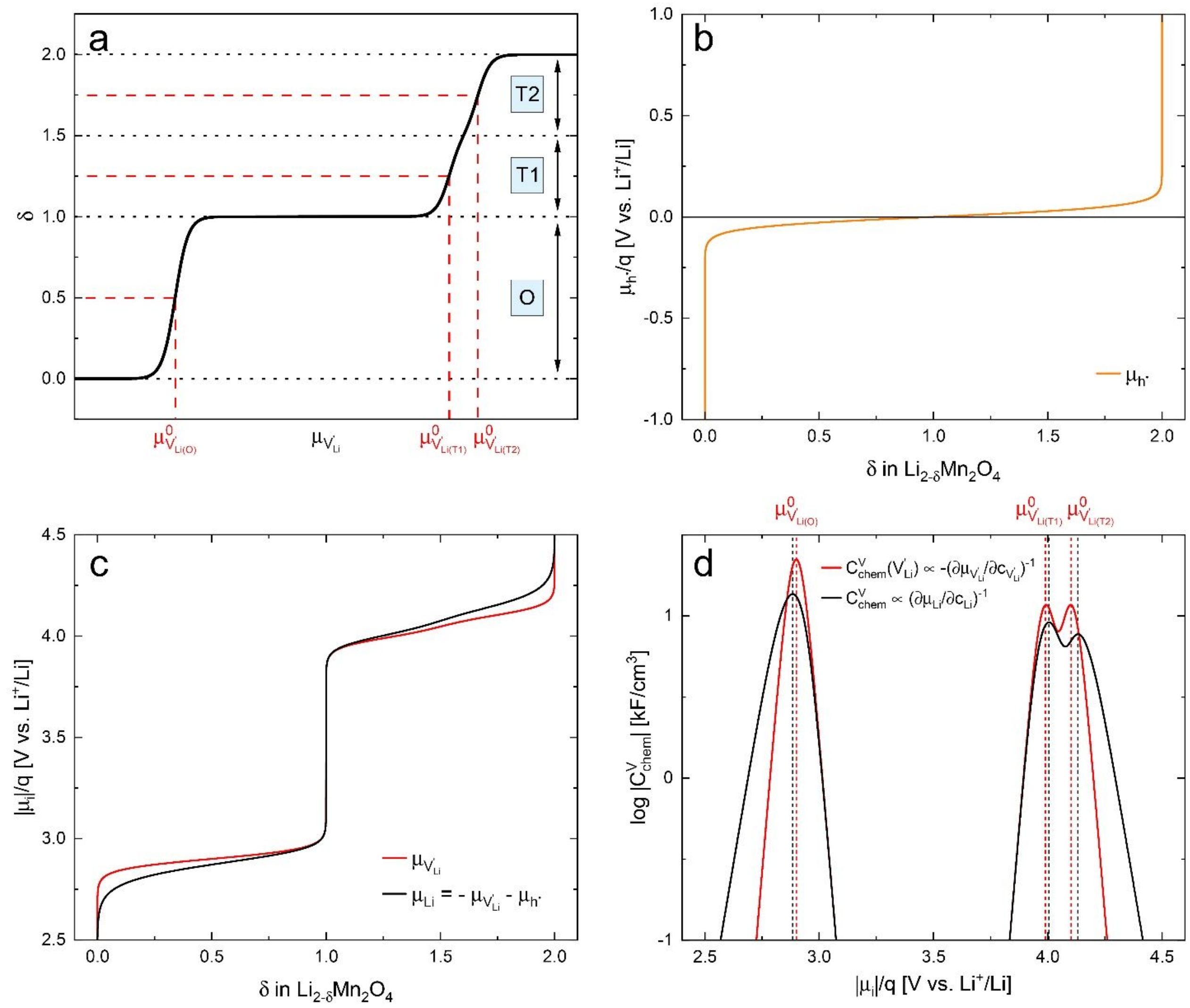

Figure S4 (a) Total nonstoichiometry δ in $Li_{2-\delta}Mn_2O_4$ as a function of the total Li vacancy chemical potential $\mu_{V'_{Li}}$, as calculated from equation S3. The functional inverse $\mu_{V'_{Li}}(\delta)$ can be obtained numerically. (b) Chemical potential of electron holes $\mu_{h^\bullet}$ as a function of δ, as calculated from equation S4. (c) Total Li chemical potential μ_{Li} (black) as calculated from equation S5, compared to $\mu_{V'_{Li}}$ (red) obtained by numerical inversion of $\delta(\mu_{V'_{Li}})$ in (a). (d) Total volume-specific chemical capacitance C^V_{chem} (black) from equation S6 compared to its isolated vacancy component $C^V_{chem}(V'_{Li})$ (red) obtained via equation S7. The peaks of C^V_{chem} are slightly shifted with respect to the inserted values of $\mu^0_{V'_{Li(j)}}$, due to the concentration-dependent contribution of $\mu_{h^\bullet}$ to μ_{Li}.

$$\delta_i = \frac{[V'_{Li(i)}]}{c^0} = \frac{y_{V'_{Li(i)}}}{1 + e^{\frac{\mu^0_{V'_{Li(i)}} - \mu_{V'_{Li}}}{kT}}} \quad \text{(S2)}$$

$$\delta = \sum \delta_i(\mu_{V'_{Li}}) = \frac{1}{1 + e^{\frac{\mu^0_{V'_{Li(0)}} - \mu_{V'_{Li}}}{kT}}} + \frac{0.5}{1 + e^{\frac{\mu^0_{V'_{Li(T1)}} - \mu_{V'_{Li}}}{kT}}} + \frac{0.5}{1 + e^{\frac{\mu^0_{V'_{Li(T2)}} - \mu_{V'_{Li}}}{kT}}} \quad \text{(S3)}$$

$$\mu_{h^\bullet} = \mu^0_{h^\bullet} + kT\ln\frac{x_{h^\bullet}}{1 - x_{h^\bullet}} = \mu^0_{h^\bullet} + kT\ln\frac{\frac{\delta}{2}}{1 - \frac{\delta}{2}} \quad \text{(S4)}$$

$$\mu_{Li} = -\mu_{V'_{Li}} - \mu_{h^\bullet} \quad \text{(S5)}$$

$$C^V_{chem} = q^2\left(\frac{\partial \mu_{Li}}{\partial c_{Li}}\right)^{-1} \quad \text{(S6)}$$

$$C^V_{chem}(V'_{Li}) = -q^2\left(\frac{\partial \mu_{V'_{Li}}}{\partial c_{V'_{Li}}}\right)^{-1} \quad \text{(S7)}$$
